# The 2018 summer heatwaves over northwestern Europe and its extended-range prediction

**DOI:** 10.1038/s41598-020-76181-4

**Published:** 2020-11-06

**Authors:** Mien-Tze Kueh, Chuan-Yao Lin

**Affiliations:** Research Center for Environmental Changes, Academia Sinica, Taipei, Taiwan

**Keywords:** Climate sciences, Atmospheric science

## Abstract

This study investigated the drivers and extended-range prediction of the mid-July to early August 2018 heatwaves over northwestern Europe, focusing on regional heatwave events over Scandinavia (SC) and Western Europe (WE). The persistent blocking regime (BL) was the most influential contributor for the 2018 heatwave over SC, and both the Atlantic Low regime (AL) and North Atlantic Oscillation (NAO) were secondary contributors for the heatwave, but with different effect directions. The major contributor to the heatwave over WE was AL. These causal relationships remained valid when the evolution of warm spells was considered. A multi-model ensemble of real-time forecasts from the subseasonal to seasonal (S2S) database captured the evolution of the warm spells over SC and WE up to 3 weeks in advance. However, the predictions of heatwave occurrence and significance for the two regions are unsatisfactory. BL and AL can be predicted 2 weeks in advance, resulting in the successful predictions of warm spells over SC and WE. Although variations in Azores High and NAO were captured in the forecasts, their contribution to the warm spells remains unclear.

## Introduction

In the summer of 2018, prolonged and severe heatwave and drought conditions occurred across northwestern Europe, leading to socio-economic and environmental impacts^1–4^. While anthropogenic climate change could be responsible for certain proportion of these record-breaking warmth and precipitation deficits, natural variability was also indispensable factor^4–6^. Previous studies have suggested several drivers for these 2018 summertime European heatwave and drought events, including persistent blocking^2,6^, subtropical ridge or tropical continental air flow^5,7^, an exceptionally strong stationary Rossby Wave-7 pattern^8,9^, prevalence of the positive phase of the summer North Atlantic Oscillation (NAO)^5,9^, the Atlantic sea-surface temperature (SST) tripole pattern^5,10^, local SST and soil moisture content^5,11^. Moreover, improved seasonal^10^ and short-term^11^ predictions have been made using reasonable representations of the SST tripole pattern and local SST/soil moisture content, respectively, for the 2018 summer dry and hot conditions. However, study of extended-range prediction for the 2018 summer European heatwaves has also been absent.

Persistent and severe heatwaves pose serious threats to health and welfare. Skillful heatwave prediction at the sub-seasonal or extended-range timescales (2–4 weeks) can allow for more time for mitigation. The exact timing of the evolution of hot extremes is also crucial for preparation. However, prediction of the occurrence of a given heatwave events over a specific location 3–4 weeks in advance remains a challenge. Slow varying drivers including Madden–Julian Oscillation, mid-latitude persistent flow regimes, and land–atmosphere interaction could be potential sources of the sub-seasonal predictability of European 2 m-temperature extremes^12–15^. Although severe and/or long-lasting heatwaves could be more predictable at the extended-range timescales, such as the 2010 Russian heatwave^14,15^, the skill of the prediction of blocking variability would largely control the forecast of the onset, duration, and amplitude of the heatwave^13^.

A reliable identification of the drivers of heatwaves can improve predictability and preparedness^16–19^. Irrespective of their location, heatwaves are commonly associated with high pressure systems and anomalous warm air masses^16–24^. In particular, most summertime European heatwaves are associated with atmospheric blocking^2,3,16,19,22,25,26^. Other persistent weather regimes over the Atlantic-European sector, such as subtropical ridge or Atlantic Low, have also been related to Western European heatwaves^16,25,27^. Extended-range prediction for European heatwave events is therefore related to the predictability sources of the atmospheric blocking or persistent weather regimes. Reliable representations of slowly evolving large-scale circulation regimes can provide additional sources of predictability^2,16,18,25^, which could lead to increase in the valid lead times for European heatwave in forecast models. The summer North Atlantic Oscillation (NAO), which is a dominant pattern in the North Atlantic—European region and reflects jet stream and storm track variability, is an important factor^28–30^. The positive phase of summer NAO, which is commonly associated with northward anomalies of jet stream and storm track, has been related to warm and dry conditions over northwest Europe^16,28–30^. The spatial pattern of positive summer NAO defined by Folland et al.^29^ is similar to the summer European blocking regime identified by Cassou et al.^25^. The positive summer NAO is also associated with a northwestern extension of the Azores high and an eastward shifted Icelandic low^29^, suggesting an inter-dependency among the variability of summer NAO, blocking, and Azores High. In addition, the North Atlantic SST^10,16,31^ and tropical Atlantic convective anomalies^25^ can also play a role in the development of European heatwaves, which indicates the potential contribution of ocean temperature anomalies. Improving the dynamical links among heatwave-related circulation regimes as well as ocean temperature anomalies in the forecast model will contribute to extending the prediction range for European heatwaves.

**Figure 1:**
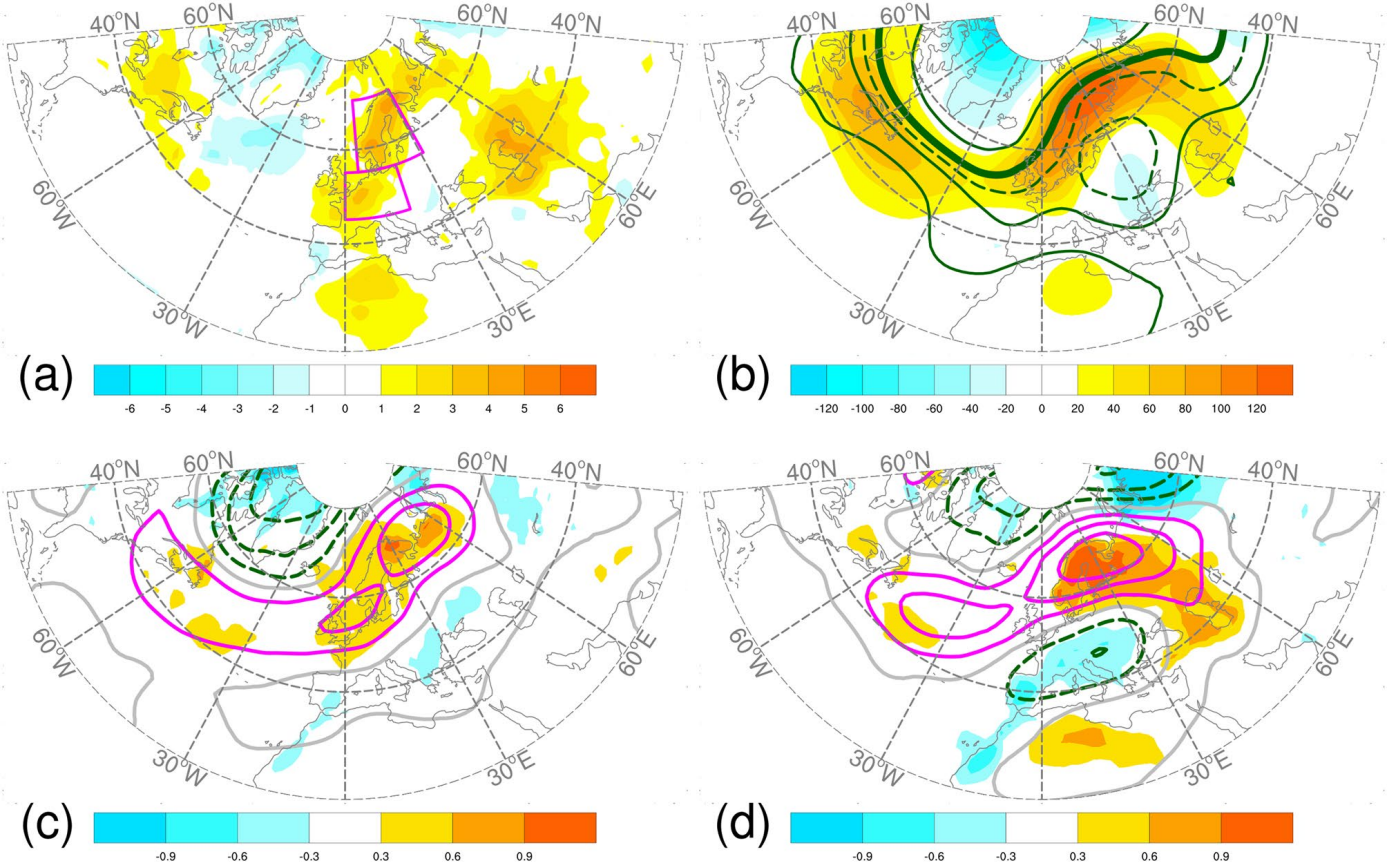
2018 July (**a**) 2-m temperature anomalies (shading, °C) and (**b**) 500-hPa geopotential height anomalies (shading, m) and absolute 500-hPa geopotential height (contour, m). The solid contours are 5600, 5700, and 5800 m, and dashed contour is 5750 m. Anomalies are relative to the period of 1999–2010. Boxes show the area used to define Scandinavia (SC) and Western Europe (WE). Regression of 2-m temperature anomalies (shading, °C) and 500 hPa geopotential height anomalies (shading, m) onto the monthly circulation indices (**c**) NAO and (**d**) SCA. Contour interval is 2; solid and dashed contours for positive and negative correlation coefficients, respectively, and gray color for zero contours. *NAO* North Atlantic Oscillation, *SCA* Scandinavia Pattern. See text for details of the data sources. The linear regression analyses were conducted over the period of July 1999–2018. The maps were generated using software NCAR Command Language (https://www.ncl.ucar.edu/)^32^ with the built-in Ncarg4_0 database.

In July 2018, a warmer than average temperature was present over Northern and Western Europe. This was associated with predominant atmospheric blocking circulation across the European continent and positive height anomalies at 500-hPa centered over northern SC (Fig. 1a,b). The major features observed in the monthly mean of July 2018 could be attributed to the atmospheric conditions during the second half of July (Supplementary Fig. S1). Among the previous studies for the European extreme weathers in the summer of 2018, none of them focused on the dynamic drivers for the heatwave event in the second half of July^1–11^. In this study, we investigated the dynamic drivers of the heatwaves over northwestern Europe that occurred from mid-July to early August 2018, with an emphasis on the regional heatwave events in Scandinavia (SC) and Western Europe (WE). The contributions of the following four circulation regimes were investigated: the Scandinavia blocking (BL), the Atlantic Low (AL), the Azores High (AZH), and summer NAO. We also analyzed the real-time forecast of S2S database with intent to verify whether the dynamic drivers of the heatwave can be approved by the extended-range prediction.

## Results

### Observation

The circulation background of 2018 summer was characterized by the persistence of Scandinavian blocking situation^2,6^ and prevalence of the positive phase of NAO^5,9^. The northern hemisphere teleconnection Scandinavia Pattern (SCA) can be seen as an indicator of the dominance of blocking situation. Here we examine the anomalous height pattern in association with the NAO and SCA using their monthly indices (see “Data and Methods” for the data sources and definitions of the two indices). Linear regressions of monthly 2-m temperature and 500-hPa height anomalies onto monthly NAO and SCA, respectively, for the preceding 20 years revealed clear relationships between near-surface heat anomalies and 500-hPa height anomalies over Europe (Fig. 1c,d). These two teleconnection patterns have some similarities. The southern node of the NAO north–south anomalous 500-hPa height dipole extended from the northwest Atlantic to northwest Europe, where surface heat anomalies are found. The positive node of the north–south 500-hPa wave train structure of the SCA, which was almost co-located with the positive 500-hPa height anomalies during July 2018, was associated with strong surface heat anomalies. The westward extension of this positive node of the SCA overlaps with the northwest Atlantic portion of the southern node of the NAO, whereas both substantially differ in their interannual variations despite their spatial similarities. The temporal linear correlation coefficient (0.24) between the two monthly indices was nonsignificant during the preceding 20 years. From June to August in 2018, the monthly NAO index had consistently positive values, whereas the SCA index had positive value in July only.

**Figure 2:**
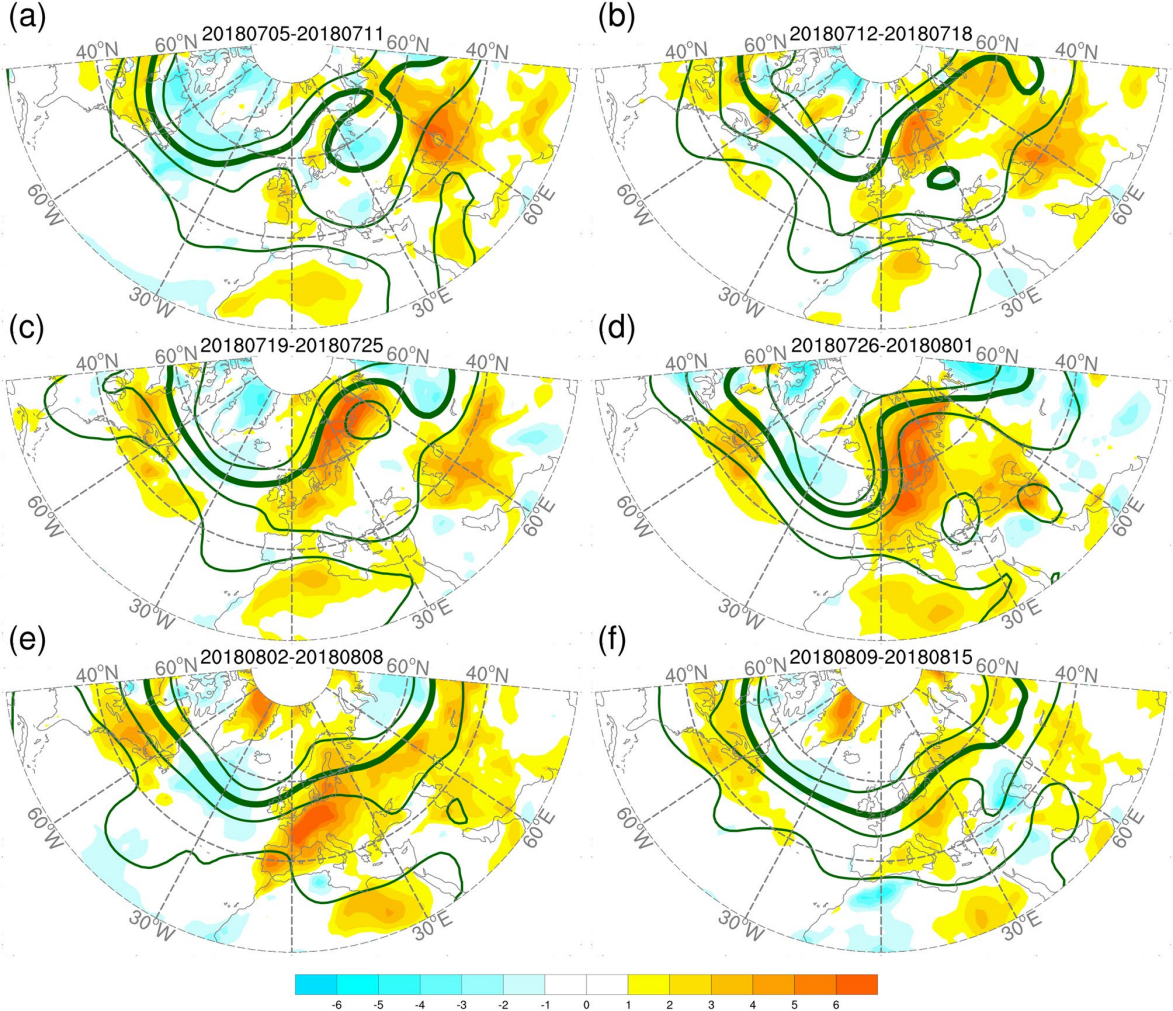
Weekly anomalies of 2-m temperature (shading, °C) and weekly mean of 500-hPa geopotential height (contour, m) for 5 July–15 August, 2018. The solid contours are 5600, 5700, 5800, and 5900 m. Anomalies are relative to the period of 1999–2010. The maps were generated using software NCAR Command Language (https://www.ncl.ucar.edu/)^32^ with the built-in Ncarg4_0 database.

From mid-July to early August 2018, a large area of heat anomalies emerged across northwest Europe. The anomalous warmth over SC and WE underwent various evolutions in regional atmospheric circulations. Our focus was on the key period from 12 July to 8 August, 2018. In the week prior to the key period, the European continent was occupied by a cut-off low at 500-hPa (Fig. 2). During the 4-week period from 12 July to 8 August, 2018, broad-scale blocking situations persisted for the first 3 weeks. During this long period of blocking dominance, the system occupied the European continent with gradual changes in the shape of the blocking ridge. The upstream trough deepened in association with an enhanced ridging situation, leading to intensifying heat anomalies across northwest Europe. The blocking ridge then rapidly decayed, and southern open ridges emerged and dominated during the fourth week, along with intensified heat anomalies over WE and reduced heat anomalies over SC. The intense heat anomalies across the region ended in the following week. The spatial patterns of heatwave over SC and WE have been identified by Stefanon et al.^33^. The heatwaves over SC and WE have also been attributed to blocking ridge and subtropical ridge, respectively, in previous studies^21,26,27,34^.

**Figure 3:**
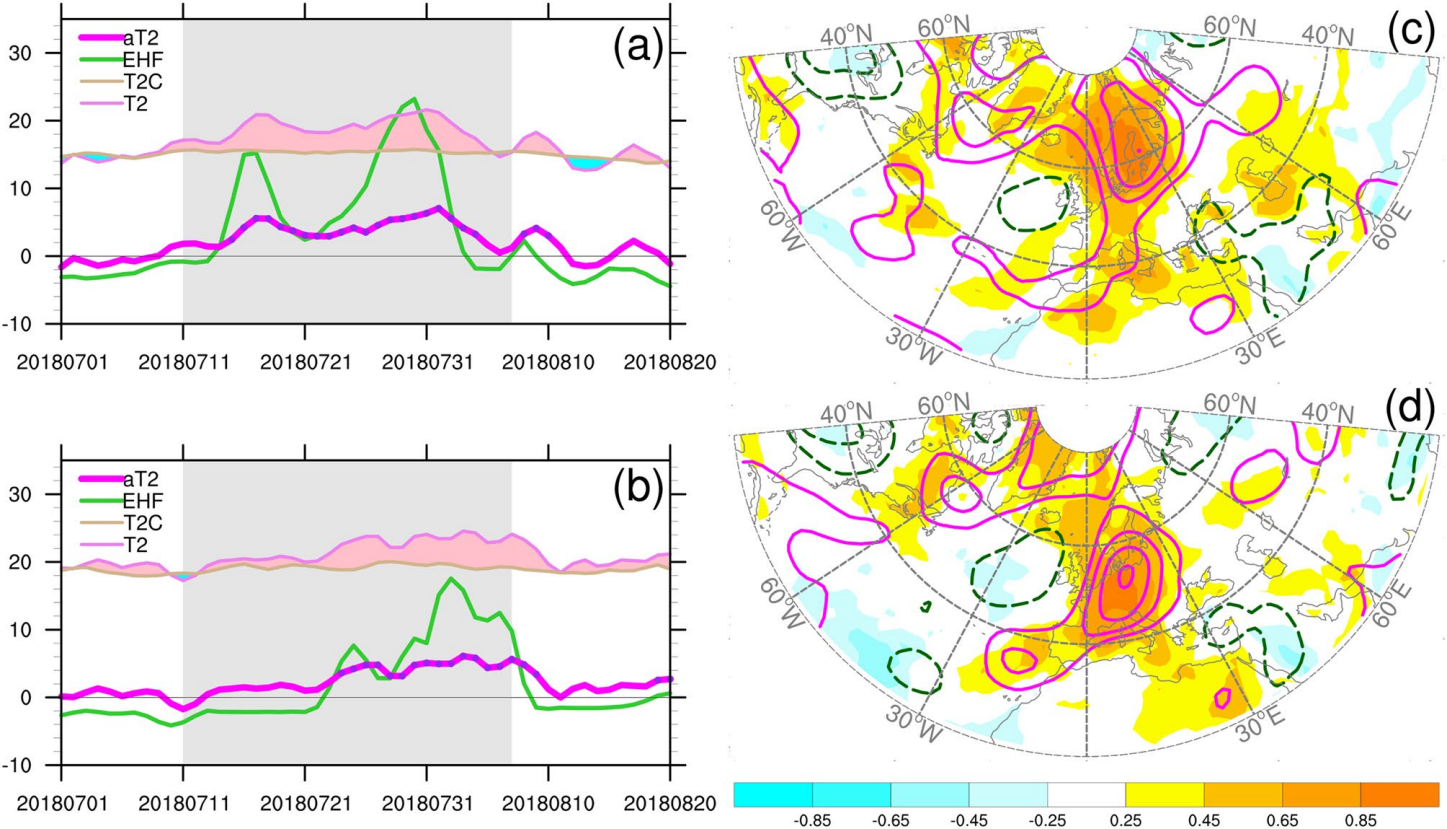
Left panels: time series of the area-averaged 2-m temperatures for (**a**) SC and (**b**) WE. The corresponding areas are defined by the boxes shown in Fig. 1a. Parameters shown are daily 2-m temperature (T2), long-term daily mean (T2C), daily 2-m temperature anomalies (aT2), and modified excess heat factor (EHF). Dots indicate the daily 2-m temperature exceed the 90th percentile threshold. Units: °C. The reference period is 1999–2010 for the calculation of aT2 and EHF. Gray shading region covers the period of 12 July–8 August, 2018. Right panels: Pearson correlation coefficients of daily anomalies of 2-m temperature (shading, °C) and 500-hPa geopotential height (contour, m) with the area-averaged temperatures for (**c**) SC and (**d**) WE. Solid and dashed contours for positive and negative correlation coefficients, respectively. Contour interval is 2, and zero contours are omitted. The correlation coefficients were calculated for period of 1 July–31 August, 2018. The maps were generated using software NCAR Command Language (https://www.ncl.ucar.edu/)^32^ with the built-in Ncarg4_0 database.

The area averaged 2-m temperatures over SC and WE exhibited different temporal evolutions. From mid-July to early August, the daily 2-m temperatures were significantly higher than their respective daily climatological temperature (Fig. 3a,b). The key period was from 12 July to 8 August, 2018—the 4-week period shown in Fig. 2. During this crucial period, the temporal evolution in SC exhibited a double peaked pattern, whereas that of WE exhibited a slower warming trend with a warm spell lasting until a few days after 8 August. For SC and WE, the area-averaged 2-m temperature anomalies (aT2) revealed that the warm spells lasted for more than 4 weeks—during which time the heatwave occurrences were identified as having durations of 21 and 17 days, respectively, based on the dates when aT2 exceeded the respective 90th percentile thresholds (T2-P90). EHF emphasis the signal of the heatwave evolution, thus providing warnings for the rapid rise in the anomalous temperature variations.

The anomalous temperature variations in the two regions were associated with different anomalous regional circulation patterns (Fig. 3c,d). Although their aT2 values were closely correlated with overlapping positive anomalous patterns in 2-m temperature and 500-hPa height, the location and shape of the anomalous patterns were different. For both regions, a dipole structure was observed with a strong positive node and a weak negative node. The dipole structure for SC exhibited a northeast–southwest orientation, reflecting persistent blocking situation during the July–August 2018 period. The dipole structure for WE exhibited an east–west orientation, resembling that for AL which has been related to summertime European heatwaves^16,25^. We also calculated the correlation coefficients of T2-P90 with the anomalous 2-m temperature and 500-hPa height; the patterns were similar to those of aT2. The results suggested that corresponding circulation indices can be constructed to relate the variations of area-averaged temperatures and circulation regimes; moreover, such indices can be used to assess the predictive performance of a model representing these relationships. On a monthly timescale, regional 2-m temperature variability across the northwest Europe was positively correlated with the variations in the NAO, SCA, and AZH indices during the preceding 20 years (Fig. 1, Supplementary Fig. S2). However, their interactions on a daily timescale remain unclear.

**Figure 4:**
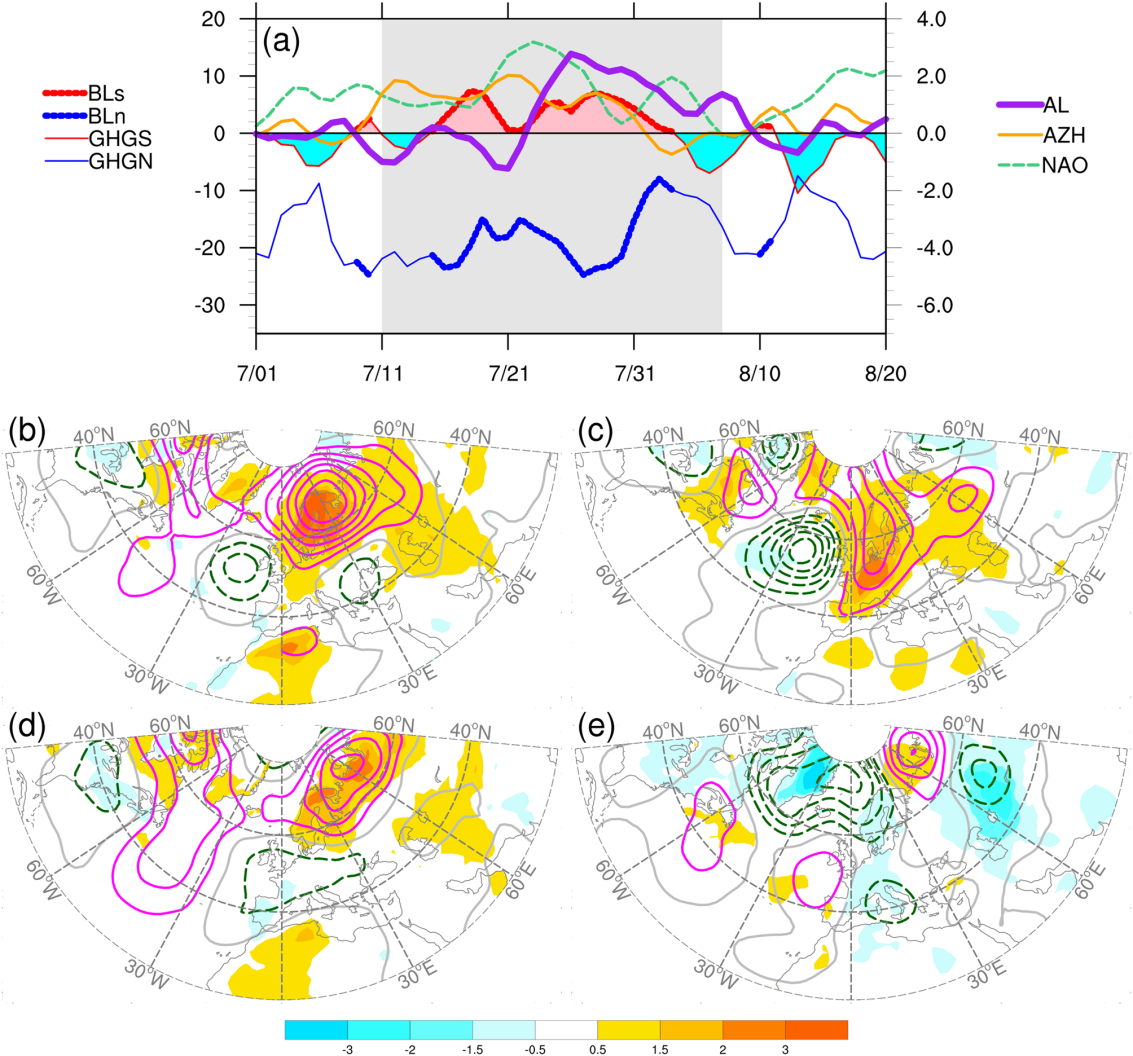
Top panel: (**a**) time series of a variety of circulation indices during period of 1 July–20 August, 2018. Indices include: GHGS and GHGN strengths for atmospheric blocking variability, Atlantic Low (AL), Azores High (AZH), and North Atlantic Oscillation (NAO). Dots denote both GHGS and GHGN strengths meet the criteria (BLs and BLn; see text in “Methods”), indicating blocked day. Gray shading region covers the period of 12 July–8 August, 2018. The GHGS strength is also termed as BL in this study. Central and bottom panels: linear regression of daily anomalies of 2-m temperature (shading, °C) and 500-hPa geopotential height (contour, m) onto the standardized circulation indices (**b**) BL, (**c**) AL, (**d**) AZH, and (**e**) NAO. Solid and dashed contours for positive and negative regression coefficients, respectively, and gray contours for zero. Contour interval is 150 m per standard deviation change in index for 500-hPa geopotential height anomalies. Contour interval for 2-m temperature anomalies is denoted by label bars, the units is °C per standard deviation change in index. The linear regression analyses were conducted for period of 1 July to 31 August, 2018. The maps were generated using software NCAR Command Language (https://www.ncl.ucar.edu/)^32^ with the built-in Ncarg4_0 database.

The daily circulation regime indices exhibited different temporal evolutions during the analysis period (Fig. 4 a). For blocking variability, the GHGS and GHGN strengths were negatively correlated. During the period of interest (i.e., 12 July–8 August), the GHGS strength reached 0 on 2 days between the two peaks, reflecting a reorganization of the blocking ridge in the first 2 weeks of this period (Fig. 2a,b). GHGS strength alone was sufficient to reveal blocking activity. Therefore, we adopted GHGS strength to represent the blocking index, which we term BL. The AL index exhibited a sharp increase on approximately 21 July, corresponding to the development of the heatwave over the WE (Fig. 3a). During the key period, AZH maintained positive values until the last week. From July to August, positive values were observed for the NAO index, except for 2 days in early August.

To understand how the 2-m temperatures and 500-hPa heights varied over the Atlantic-European sector in association with these indices, we conducted linear regressions for the two anomalous fields onto these standardized indices (Fig. 4b–e). The regression maps of standardized BL and AL reveal patterns similar to those in Fig. 3c,d; BL exhibited a considerably stronger positive node, and AL exhibited a stronger negative node and a northward shift in the positive node. The regression map for the standardized AZH also exhibited a pattern similar to that observed that for BL, with a weaker and eastward-extending negative node. The regression coefficients revealed that single standard deviation changes in BL and AZH were associated with local changes of 1.5–3 °C in heat anomalies over SC. By contrast, a single standard deviation change in AL was associated with a local change of > 2 °C in heat anomalies over WE. However, the standardized NAO was associated with a widespread area of cold anomalies over the European continent. A similar pattern was observed when we use the daily NAO index obtained from the CPC to calculate the regression coefficients (Supplementary Fig. S3).

The regression maps for BL, AL, and AZH exhibited positive 500-hPa height anomalies over SC, implying that the three indices could have been interdependent during this period. To examine the interdependence between two or more indices, we analyze the bivariate correction coefficients and partial correlation coefficients among these circulation indices. R_X–Y_ and R_X–Y(Z)_ denote the bivariate and partial correction coefficients between the indies X and Y, respectively; indices that are held constant for the partial correlation are written with the subscript Z. The bivariate correlation coefficients R_BL-AL_ and R_BL-AZH_ were both positive (> 0.4), and the result was statistically significant (Table 1). The results of all other bivariate correlations were nonsignificant. We then used the second-order partial correlation coefficient to inspect the actual variance explained by each pair of circulation indices when the influences of the other indices were all eliminated. The second-order partial correlation coefficients R_BL-AL(AZH,NAO)_ and R_BL-AZH(AL,NAO)_ exhibited a slight increase compared with their respective bivariate correlation coefficients, indicating a direct relationship of BL with AL or AZH. The absolute value of R_AL-AZH(BL,NAO)_ was higher than that of R_AL-AZH_, and the result for R_AL-AZH(BL,NAO)_ was significant, whereas that of R_AL-AZH_ was nonsignificant. Therefore, the negative relationship between AL and AZH emerged when other circulation regimes were held invariant. The first-order partial correlation coefficients revealed that NAO had no direct effect on the relationships among the other three indices. Accordingly, the positive correlations of AL and AZH with BL strongly affected the negative correlation between AL and AZH.

**Table 1:** Correlation matrix for the T2-P90, BL, AL, AZH, and NAO.

	Variables	BL	AL	AZH	NAO
Bivariate correlation coefficient between indices	AL	0.407**			
	AZH	0.469**	− 0.183		
	NAO	0.015	− 0.175	0.184	
Bivariate correlation coefficient between T2-P90 and indices	SC(T2-P90)	0.803***	0.497***	0.392**	− 0.221
	WE(T2-P90)	0.212	0.756***	− 0.165	− 0.234
Second-order partial correlation coefficient between indices	AL	0.563***			
	AZH	0.601***	− 0.442**		
	NAO	0.000	− 0.121	0.125	
Third-order partial correlation coefficient betweenT2-P90 and indices	SC(T2-P90)	0.664***	0.352**	0.270*	− 0.390**
	WE(T2-P90)	− 0.171	0.717***	0.091	− 0.156

Numbers shown are the corresponding bivariate and second-order and third-order partial correlation coefficients. The second-order partial correlation coefficients are between two indices when all other two indices are held constant. The third-order partial correlation coefficients are between the T2-P90 over SC or WE and one of the indices when all other three indices are held constant. The correlation coefficients were calculated using Pearson method. The sample size is 62. The statistical significance is assessed with two-tailed Student’s *t* test, the p values that < 0.001, < 0.01, and < 0.05 are denoted by ***, **, and *.

The interdependency between BL, AL, and AZH can be interpreted as follows. BL and AL both explained the variability in anomalous ridging activities over northwest Europe, leading to a positive correlation for the period of blocking dominance. The positive correlation between BL and AZH indicated a connection between the subtropical and high-latitude regions. An enhancement of the Azores High may result in an increase in the meridional gradient of the 500-hPa height, leading to changes in the jet stream and storm activities, which may facilitate baroclinic development for the growth of the blocking ridge. The development of AL accompanied by a decline in AZH index values may be a result of a westward shifting or weakening of the Azores High. All these interpretations are based on the linear relationship between these indices. Evidence for the dynamical processes involved in these relationships is limited. However, the linear regression maps suggest that these indices can provide auxiliary information for the development of warm spells or heatwaves over SC and WE in the summer of 2018.

To address this issue, we analyze the bivariate correction coefficient and partial correlation coefficients of T2-P90 with these circulation indices. R_X_ and R_X(Z)_ denote the bivariate and partial correction coefficients, respectively, of index X with T2-P90; the indices that are held constant for the partial correlation are written with subscript Z. Over SC, R_BL_, R_AL_, and R_AZH_ were highly positive, and R_BL_ and R_AL_ were highly positive for WE (Table 1). The third order partial correlation coefficients for SC were lower than their respective bivariate correlation coefficients, but they remain positive and significant, indicating that the variation in T2-P90 was directly correlated with the change of these circulation regimes. This was only true for the R_AL(BL,AZH,NAO)_ in WE. The first-order partial correlation coefficients R_AZH(BL)_ for SC was − 0.028, considerably lower than R_AZH_ for SC. Therefore, the relatively high value of R_AZH_ for SC was strongly controlled by the linear relationship between AZH and BL, the influence of AZH on T2-P90 over SC was negligible. The correlation of NAO to T2-P90 over SC is less clear. The absolute value of R_NAO(BL,AL,AZH)_ was higher than that of R_NAO_, and R_NAO(BL,AL,AZH)_ was significant whereas R_NAO_ was nonsignificant. This indicated that a weak negative relationship between NAO and T2-P90 emerged over SC when all other circulation regimes were held invariant.

**Figure 5:**
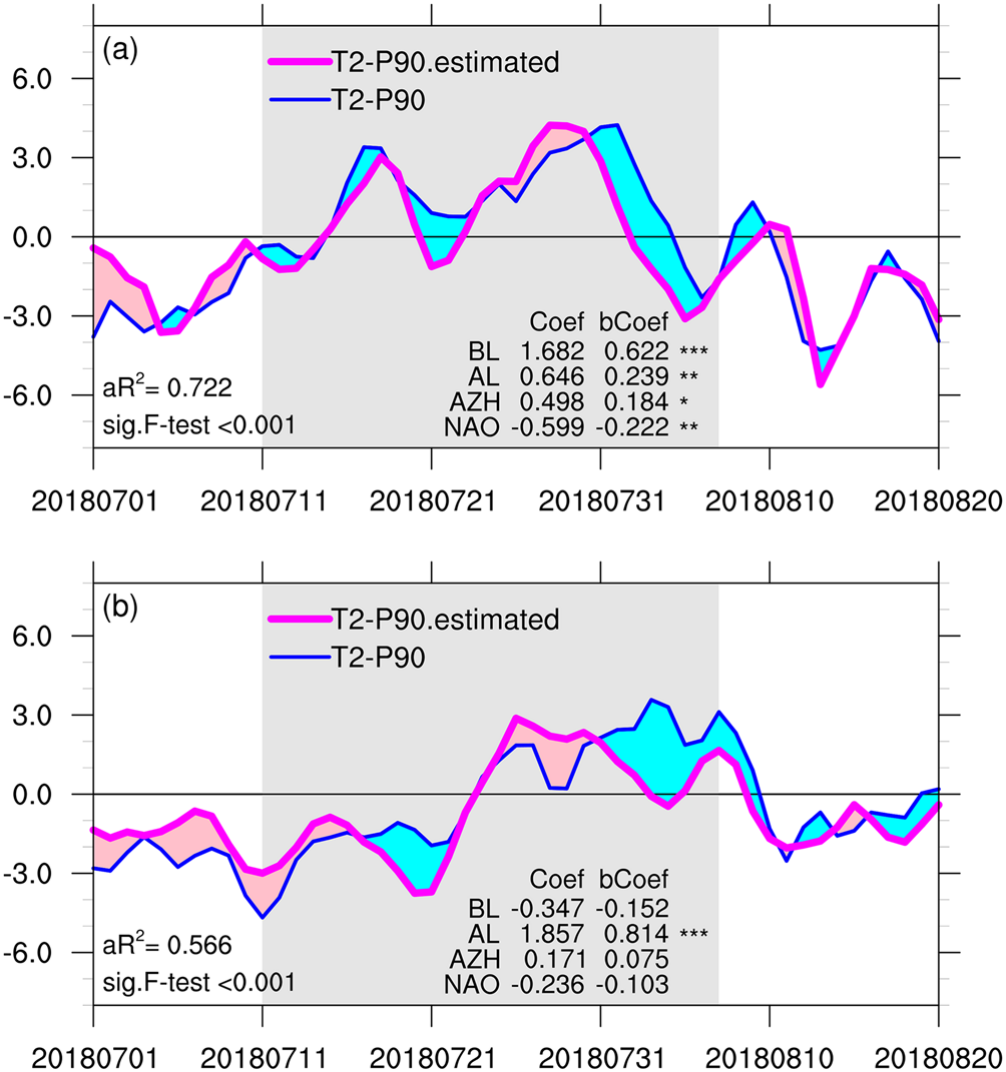
Time series of the area-averaged 2-m temperature anomalies derived from re-analysis (aT2, blue line) and estimated by multiple linear regression (magenta line) for (**a**) SC and (**b**) WE. The area-averaged 2-m temperature anomalies were regressed onto four standardized circulation indices: BL, AL, AZH, and NAO. aR^2^ denotes the adjusted coefficient of determination, and sig.F-test denotes the *p* value of *F* test for the multiple linear regression model. Coef and bCoef denote the partial regression coefficient and beta coefficient (standardized partial regression coefficient) for each index, respectively. With a sample size of 62, a 2-tailed significance is assessed with standard Student’s *t* test, the p values that < 0.001 and < 0.01 are denoted by *** and **. The multiple linear regression analyses were conducted for the period of 1 July–31 August, 2018. Gray shading region covers the period of 12 July–8 August, 2018.

We then applied multiple linear regression approach to reconstruct the area-averaged temperature in the two regions. The area-averaged T2-P90 values for SC and WE were regressed onto the four standardized circulation indices, namely the daily BL, AL, AZH, and NAO as shown in Fig. 4. Figure 5 illustrates the time evolutions of the estimated values of T2-P90 for SC and WE, along with the corresponding statistics from the multiple linear regression models. For both regions, the regression models exhibited good fit with the original time series, with adjusted R^2^ values of 0.72 and 0.57 for SC and WE, respectively. Therefore, the regression model using all four indices explained approximately 72% and 57% of the area-averaged temperature variance for SC and WE, respectively. Both results were statistically significant according to the *F* test (P < 0.001). T2-P90 over SC was significantly and positively related to standardized BL and AL and significantly and negatively related to standardized NAO (two-tailed Student’s *t* test; P < 0.01). Over WE, T2-P90 was significantly and positively related to standardized AL, whereas other indices had less significant contribution.

With other indices held constant, T2-P90 increased by 1.6 °C and 1.8 °C for a single standard deviation change in BL and AL, respectively, over SC and WE. As indicated by the beta coefficients, the most influential contributions were associated with BL and AL for the variations of T2-P90 over SC and WE, respectively. However, for SC, the partial regression coefficients of AL and NAO were also statistically significant, suggesting that their relationship with the T2-P90 over the region as well as the interdependence between these circulation indices potentially contribute to the daily variance of the aT2 over SC. In addition, AZH was positively related to T2-P90 over SC at 0.05 level of significance. Based on the partial correlation analysis, we consider the direct contribution of AZH was negligible.

When only BL and AL were used for the regression of T2-P90 over SC and WE, respectively, the linear regression model explained approximately 64% and 57% of the area-averaged temperature variance. The temporal evolutions of the estimated T2-P90 values were similar to those of their respective multiple linear regression models with all four indices. The univariate BL_T2-P90 linear regression model accounted for 64% of the variation in T2-P90 over SC, which was approximately 88% of the variation explained by the multiple linear regression model with all four indices. Overall, BL and AL were clear influences of the variations of T2-P90 over SC and WE, respectively. AL and NAO were secondary contributors to T2-P90 over SC, but with different effect direction.

The positive phases of BL and AL regimes involved positive geopotential height anomalous ridging effects over SC and WE, respectively, leading to reasonable causal interpretation for the corresponding heat anomalies. The AZH was used to measure the variability of the southern pole of NAO, however, no clear relationship was observed between the two indices. During this period, the variability of NAO was characterized by the daily fluctuation of the persistent NAO positive phase. The negative relationship between T2-P90 over SC and NAO is possibly related to short-term changes in stronger-than-normal middle latitude westerlies across the North Atlantic. The linear statistical analyses can only interpret the changes in the circulation regimes associated with changes in T2-P90 separately.

### Forecasts

We assessed whether the S2S models captured the heatwaves and corresponding circulation regimes by using the following methods. The period from 5 July to 15 August was divided into 6 weeks. For each week, the real-time forecasts were collected based on their lead times, namely the lead weeks 1–4. Therefore, 2065 forecasts were provided within each week for each lead-week. We then performed several statistical analyses of the MME for each week based on these 2065 forecasts.

**Figure 6:**
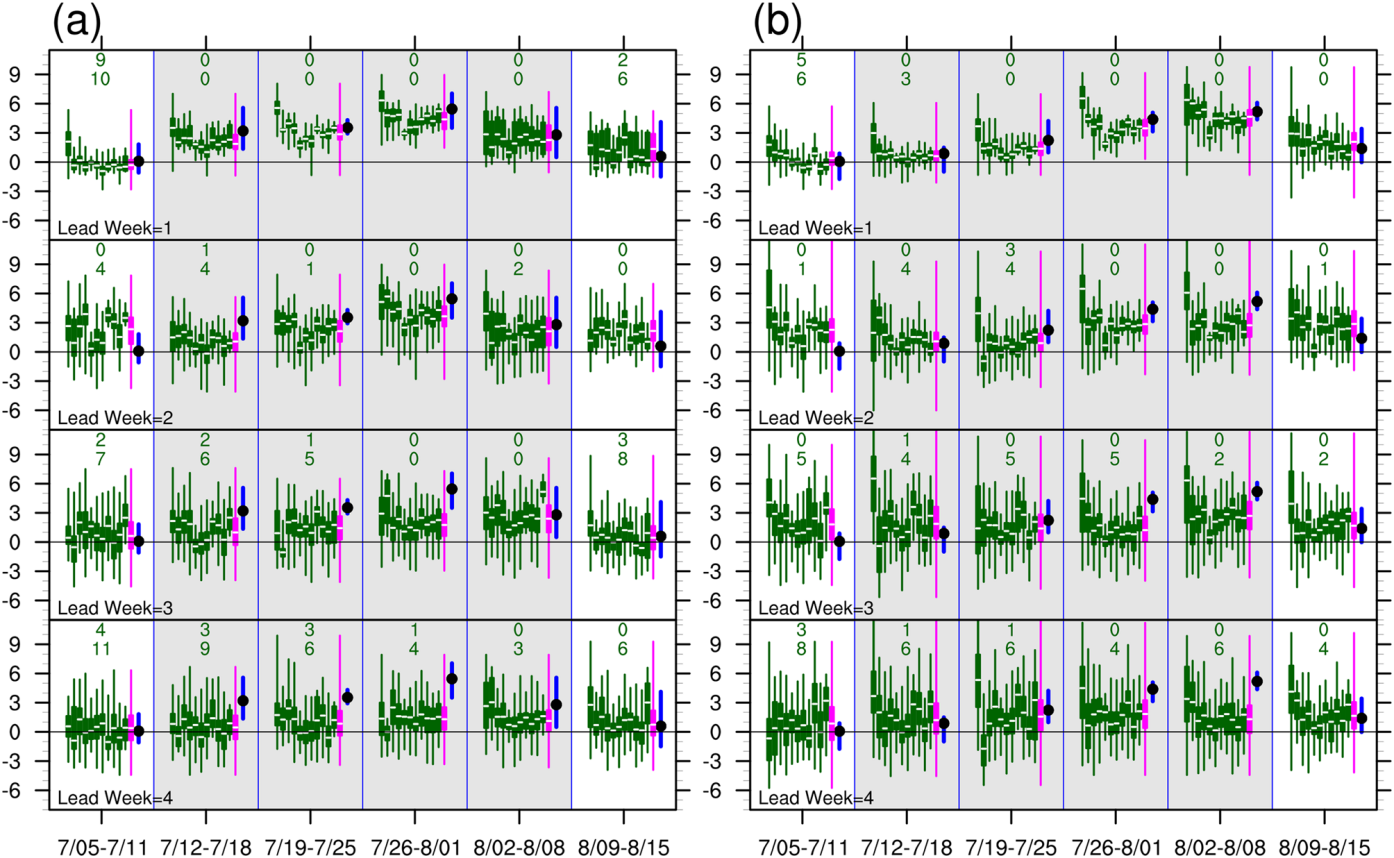
Box and whisker plots of the forecast values of area averaged daily 2-m temperature anomalies for a 6-week period during 5 July–15 August, 2018. The quartiles were derived from area-averaged daily 2-m temperature anomalies for (**a**) SC and (**b**) WE. From top to bottom panels, forecast values were obtain from lead-weeks 1 to 4. The box and whisker plots show the lower quartile (Q1), median (Q2), upper quartile (Q3), and minimum/maximum values of daily 2-m temperature anomalies in each week. Green and magenta represent forecast values from each model and multi-model ensemble, respectively. Blue thick bar indicates the range of daily 2-m temperature anomalies (from minimum to maximum) from reanalysis and black dot indicates the mean value in each week. Green numerals in each week indicate the number of models with negative values of Q2 (upper numeral) and Q1 (lower numeral). Models analyzed are listed in Supplementary Table S1. Gray shading region covers the period of 12 July–8 August, 2018.

First, we make an observation on the distribution and temporal evolution of aT2 forecasts for the 11 S2S models and the MME (Fig. 6). Our focus was on the key period from 12 July to 8 August. The box and whisker plots allow us to use the lower quartile (Q1) and median (Q2) metrics to measure the probability of a warm week for each set of forecasts, and make a rough comparison among the MME and S2S models. Positive values of Q1 and Q2 indicated that 75% and 50%, respectively, of the forecasts exceeded 0. Because a positive aT2 value indicated a warm day, a high proportion of the forecast values within a week exceeding zero indicated a high probability of a warm week. We also counted the number of models whose Q1 or Q2 values were negative. Thus, a smaller number count indicated a higher probability of a warm week in the MME.

Increasing the lead time degraded the performance of aT2 forecasts for SC and WE. For lead week 1, all models predicted above-average Q1, and the majority of the models predicted Q2 values comparable to the weekly mean of observed aT2. The temporal evolutions of the warm spell over SC and WE were accurately predicted. The magnitudes of the predicted aT2 decreased with increasing lead time, despite a large proportion of above-average aT2 forecasts. For lead week 4, the chances of a warm week remained high; most models did not differentiate warmth in the key period from that in the weeks before and after. Therefore, most models failed to predict specific warm spell 4 weeks ahead.

**Figure 7:**
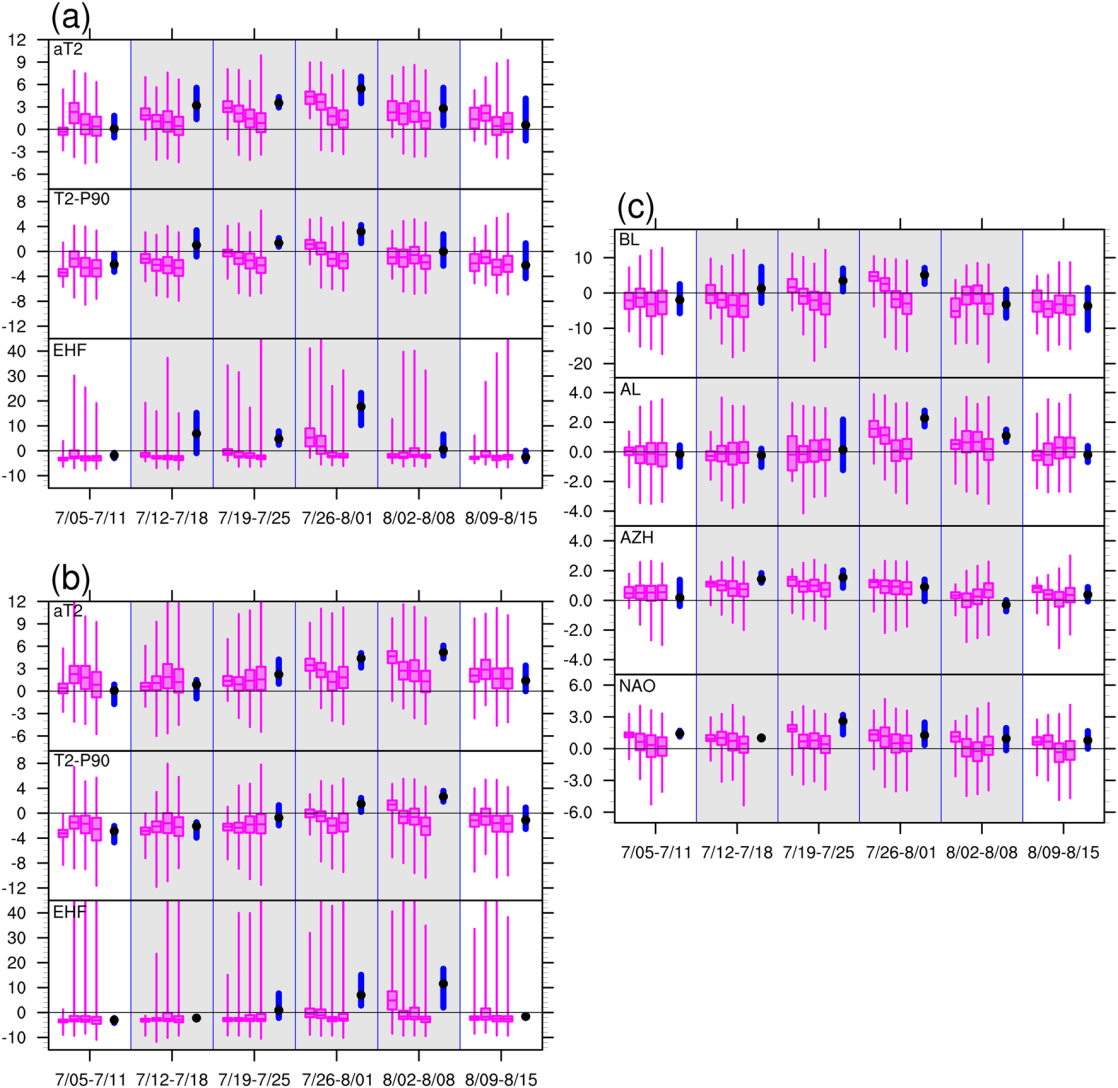
Box and whisker plots for multi-model ensemble forecast values of area-averaged daily (**a**) 2-m temperature anomaly (aT2) and deviation from the 90th percentile value (T2-P90), and excess heat factor (EHF) for SC, (**b**) same as (**a**) but for WE, and (**c**) BL, AL, AZH, and NAO for 6 weeks during 5 July–15 August, 2018. The box and whisker plots show the lower quartile (Q1), median (Q2), upper quartile (Q3), and minimum/maximum values of the respective daily forecast anomalies in each week. Within each week, the box and whisker plots represent the quartiles for lead-weeks 1–4 (from left to right). Blue thick bar indicates the range from minimum to maximum and black dot indicates the mean value for the corresponding anomalies derived from reanalysis. Gray shading region covers the period of 12 July–8 August, 2018.

Figure 7 summaries the distribution of the MME predictions. The progressive degradation of aT2 forecasts performance with increasing lead time is clearly indicated by the four descending box and whisker plots representing lead weeks 1–4. Overall, the MME predicted the occurrence of warm spells up to 3 weeks in advance. The temporal evolutions in SC and WE were captured, with forecasts for lead weeks 1–2 achieving relatively high values of Q1 in the peak weeks, namely the third week (26 July–1 August) for SC and the fourth week (2–8 August) for WE. The predictions of heatwave occurrence (T2-P90) and heatwave significance (EHF) were poor. Valid predictions were only available for SC and WE during peak weeks with lead times of 2 weeks for SC and 1 week for WE.

The lead times for valid predictions of BL and AL were short. BL was only be captured 1–2 weeks ahead for the second and third weeks (19 July–1 August), during which time the observed BL reached its mature phase. AL was captured 2–3 weeks ahead for the third and fourth weeks (26 July–8 August), which was also the time when the observed AL reached its mature phase. The MME forecasts accurately captured the evolution and magnitude of AZH. The predictions of NAO were less successful than those of AZH, however the persistence of positive phases was still captured.

**Figure 8:**
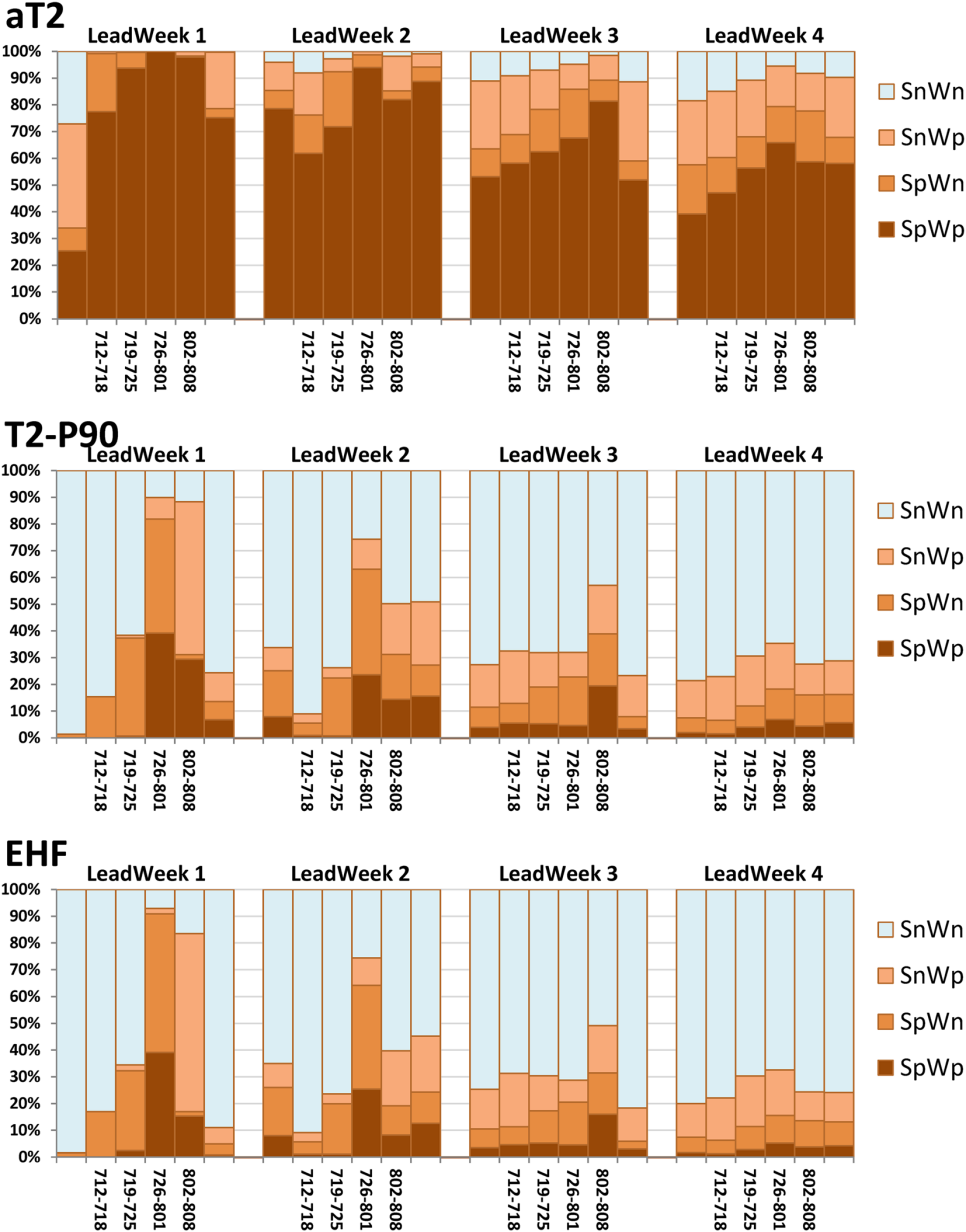
The proportions of the forecasts of the heatwave metrics (aT2, T2-P90, and EHF) as separated by their probabilities for 6 weeks during 5 July–15 August, 2018. These four possibilities are denoted as SpWp, SnWn, SpWn, and SnWp, respectively (see text for detail). All values of the proportion were calculated as a fraction of the whole, which is the total number of members from the multi-model ensembles within a week.

Although the MME did not accurately predict the heatwave occurrence, warm spells over SC and WE were successfully captured. However, the quartile statistics-based analysis did not distinguish the positive and negative phases of the circulation indices associated with the warm spell (Fig. 7). For any of three heatwave metrics, there are 4 possibilities for each forecast member at a single forecast time: the forecast values for SC and WE were either both positive or both negative, or only one of them was positive. Here we use SpWp, SnWn, SpWn, and SnWp, respectively, to denote the four possibilities. We expect to find different preferential circulation patterns in association with these four possibilities. Based on the four possibilities, the ensemble members of the MME were then stratified separately according to their daily aT2, T2-P90, and EHF forecasts for each week. For each heatwave metric, the proportion of each possibility was calculated as a fraction of the whole (i.e., the total number of members from the multi-model ensembles within a week). The results are shown in Fig. 8.

**Figure 9:**
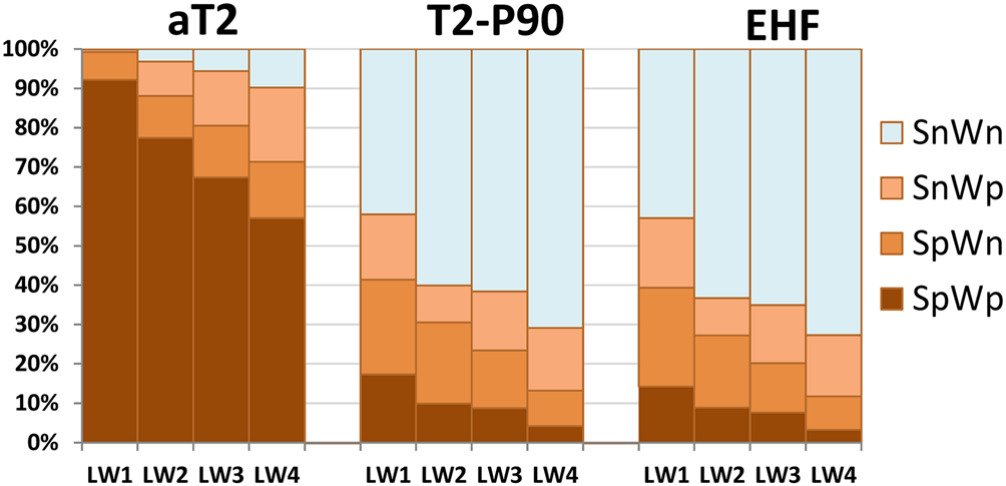
Same as in Fig. 8, but for proportions calculated during the key period from 12 July to 8 August, 2018.

High proportions of SpWp were observed in the aT2 forecasts for all the lead weeks, indicating that the MME predicted a prolonged warm spell over the region up to 3–4 weeks ahead. However, the proportions of SpWp in the T2-P90 and EHF forecasts were substantially lower than those in the aT2 forecasts. For lead weeks 1–2, both metrics can effectively distinguish heatwave occurrence of SC from that of WE by the variation in the proportions of SpWn and SnWp. The fourth week (2–8 August) for lead week 3 was still predicted to have higher proportions of positive values for both of and either of SC and WE. For lead week 4, a rather flat distribution of the proportions of positive forecasts was observed. Figure 9 summarize the proportions of these four possibilities during the key period (from 12 July to 8 August) for all heatwave metrics. From lead week 1 to 4, the decreases in the proportions of SpWp were apparent, and the variations in SpWn and SnWp were less consistent. Overall, lower occurrence of heatwaves over the SC and WE were predicted for longer lead time.

**Figure 10:**
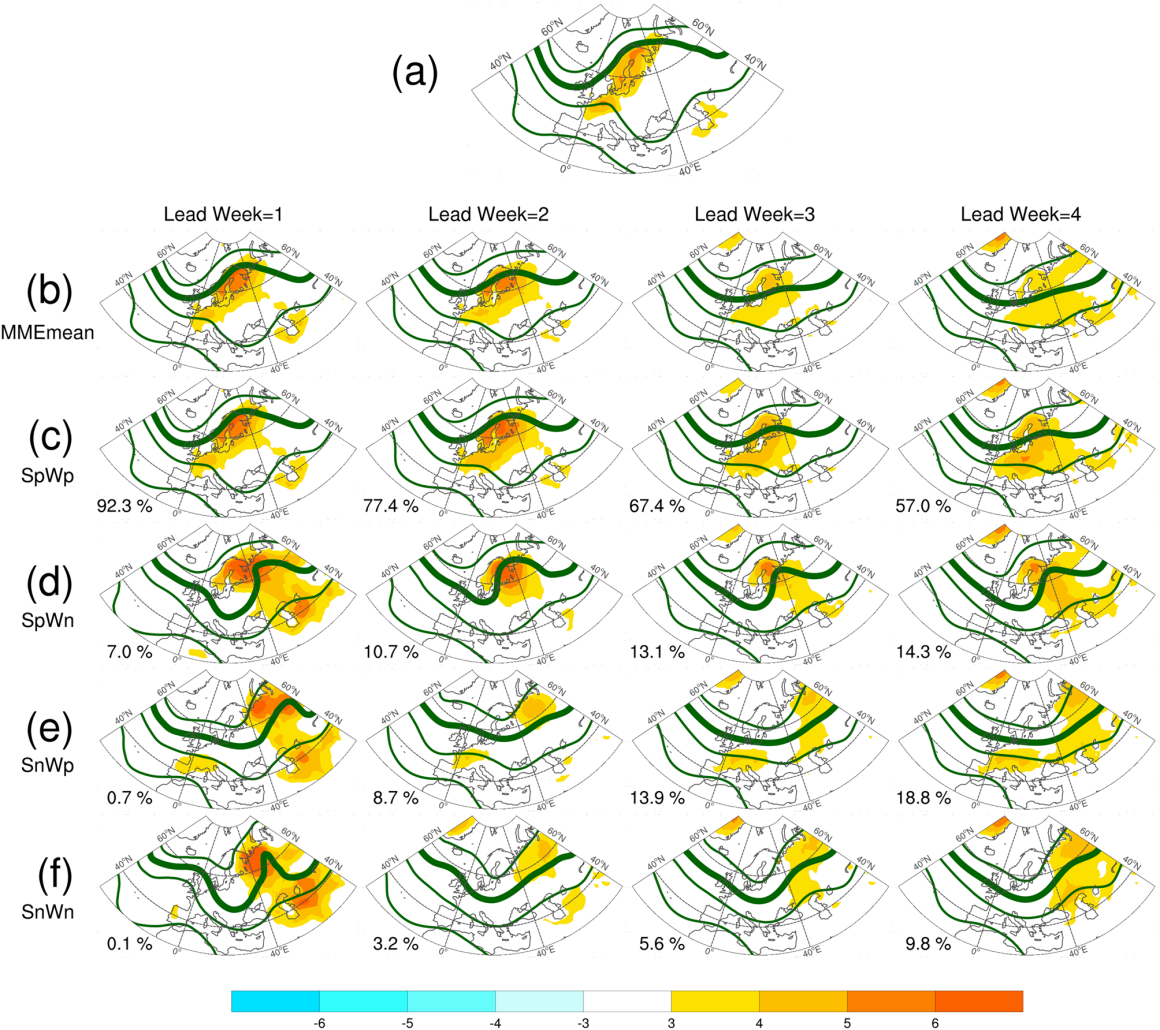
The mean anomalies of 2-m temperature (shading, °C) and mean of 500 hPa geopotential height (contour, m) for the key period from 12 July to 8 August, 2018. (**a**) For the ERA-I, (**b**–**f**) are ensemble mean calculated according to the four groups of probabilities based on the forecasts of aT2. Numeral on each panel indicates the proportions of each possibility for each lead week, also shown in this figure. The solid contours are 5600, 5700, 5800, and 5900 m. Anomalies are relative to the period of 1999–2010. The maps were generated using software NCAR Command Language (https://www.ncl.ucar.edu/)^32^ with the built-in Ncarg4_0 database.

**Figure 11:**
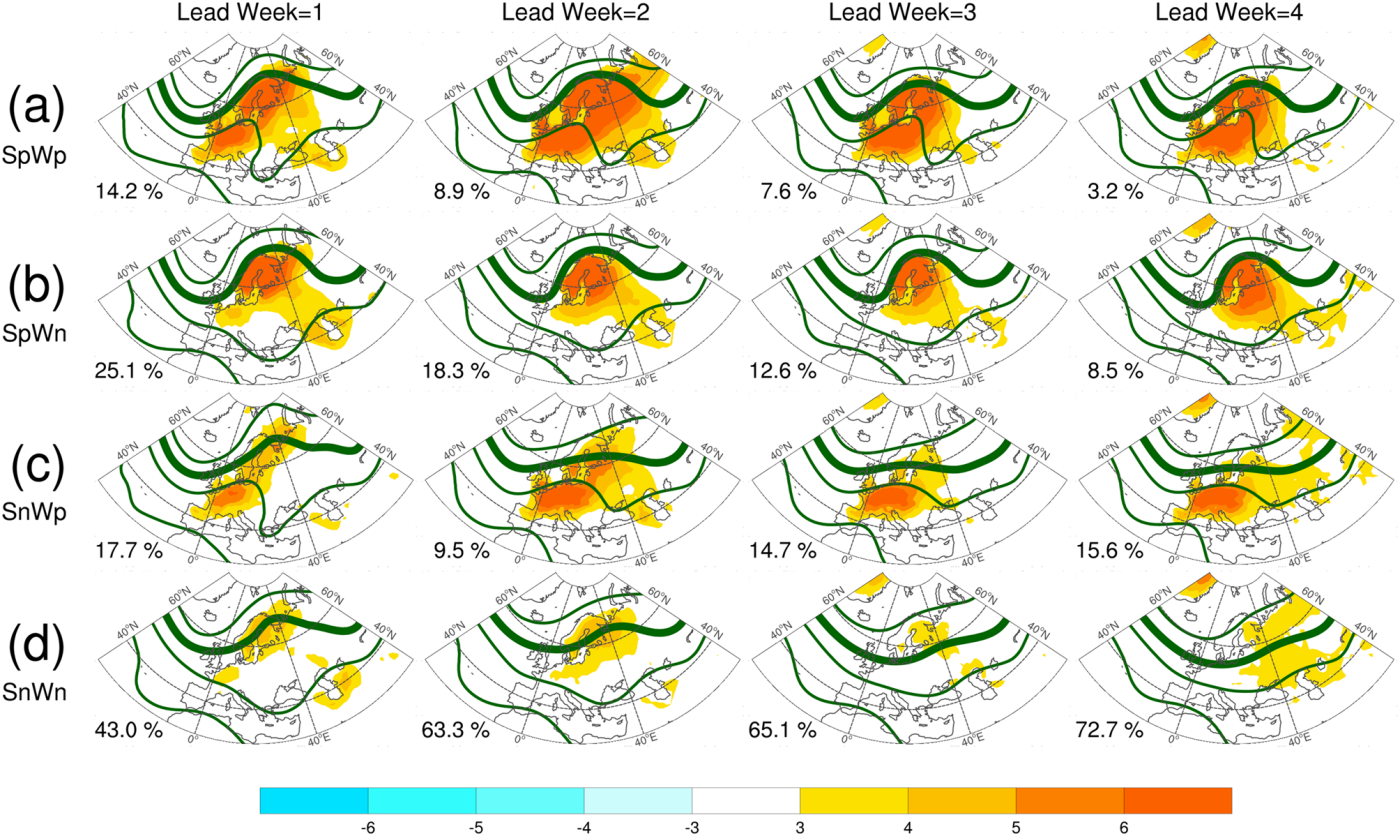
Same as in Fig. 10c–f, but for the four groups of probabilities based on the forecasts of EHF.

Figures 10 and 11 show the preferential circulation pattern during the key period in association with each of the four possibilities for aT2 and EHF, respectively. The corresponding circulation patterns for T2-P90 are highly similar to that for EHF, thus, only the results of aT2 and EHF are presented. For the first two lead weeks, the MME mean presented similar anomalous warmth over SC and WE, along with broad-scale blocking situation, as compared with the reanalysis (ERA-I). The anomalous warmth decreased and the blocking situation vanished in the forecasts of lead weeks 3–4. Because of the substantial high proportions of SpWp found in aT2 forecasts, the corresponding patterns of anomalous warmth and circulation were similar to those of MME mean. The anomalous warmth over SC alone (i.e., SpWn) were associated with a blocking situation aloft and an upstream trough, which contributed to the absence of anomalous warmth over WE. The anomalous warmth over WE alone (i.e., SnWp) were relatively weak, and were associated with weak subtropical ridge. A broad-scale 500-hPa trough was responsible for the absence of anomalous warmth over both SC and WE. Overall, the aforementioned anomalous warmth and circulation patterns are less evident for the forecasts of lead weeks 3–4. The main features of the anomalous warmth and preferential circulations classified based on EHF forecasts were generally similar to those from aT2 forecasts, but with larger amplitudes. The difference between SpWn and SnWp were larger than that found from aT2. Although the blocking situations were remarkable for the forecasts from lead weeks 1–4, the proportions of SpWp and SpWn were substantially lower than that of SnWn (see the numeral on plots, and Fig. 9). Because of this small proportion of forecasts with blocking situation, the MME forecasts of BL dropped apparently for lead weeks 3–4 (Fig. 7).

We further examined the 500-hPa height anomalies in association with these four groups of possibility (Supplementary Figs. S4, S5). The 500-hPa height anomalies associated with SnWp and SpWp as derived from EHF (also form T2-P90; not shown) exhibited an positive AL-like pattern for the forecasts of the first two lead weeks. This result reflected the findings from observation analyses of observation: positive BL and AL were major contributors to warm spell and heatwave in SC, whereas only positive AL was a major contributor to warm spell and heatwave in WE. The conditions of AZH and NAO cannot be identified visually from the 500-hPa circulation patterns or anomalies. Because AZH and NAO were mostly positive, they were not able to provide further information about the positive and negative values of aT2 and EHF.

The results presented in Figs. 10 and 11, as well as Figs. S4 and S5, suggested that the positive phases of BL and AL were preferential conditions for the predicted warm spell and heatwave in SC and WE, respectively. Accordingly, the MME forecasts captured the major relationship of the circulation regimes with the warm spell and heatwave in SC and WE based on a relatively shorter lead time. For longer lead times, the dynamic contributors for warm spell in MME remain unclear, suggesting a large model spread and uncertainty for the anomalous warmth. However, BL and AL were still the major contributors for heatwaves despite the apparent low occurrence.

**Figure 12:**
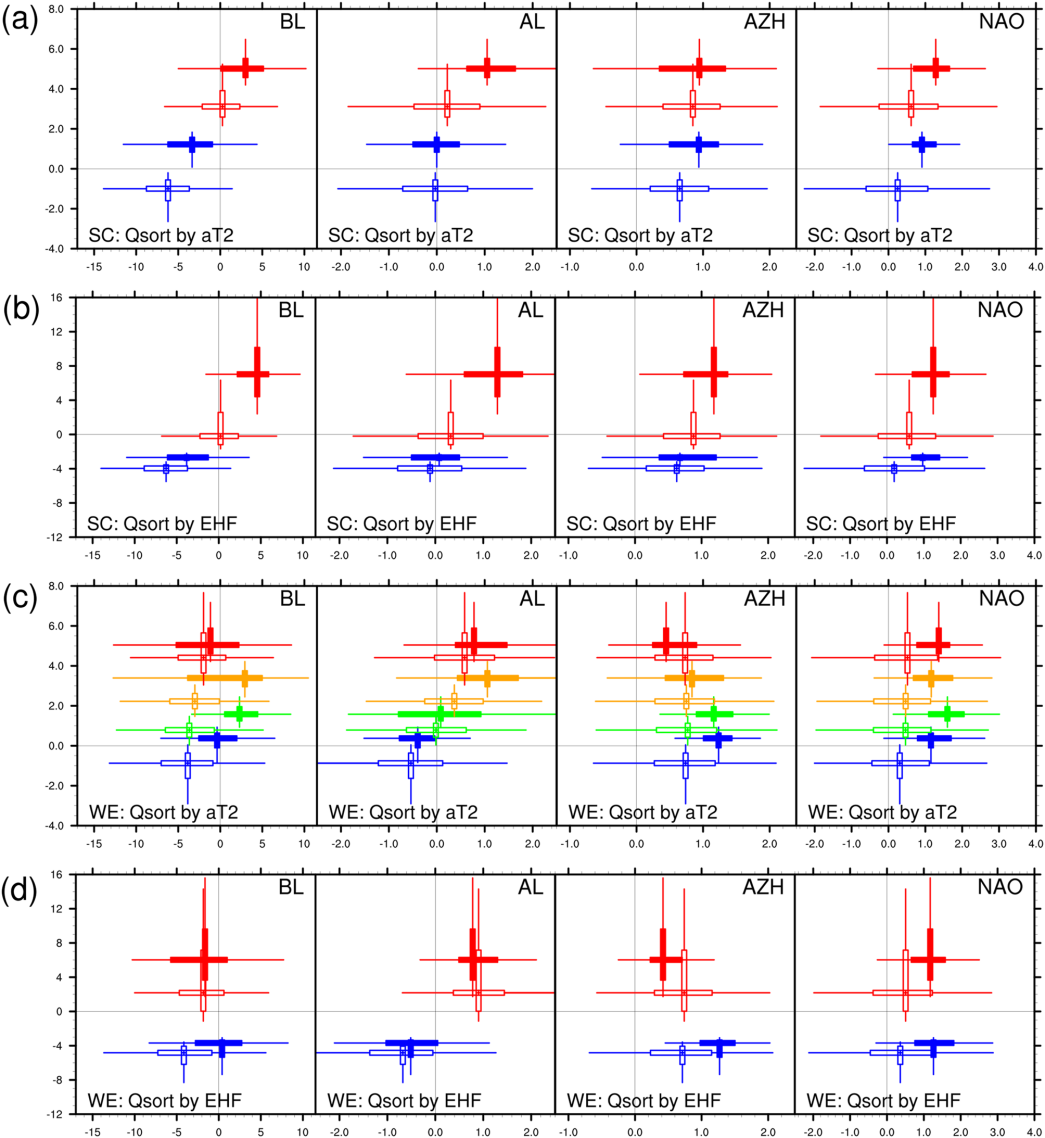
Scatter plots for the predicted 2-m temperature anomalies (aT2) or EHF against circulation indices: BL, AL, AZH, and NAO (from left to right). In each panel, the box and whisker plots show the lower quartile (Q1), median (Q2), upper quartile (Q3), and the outliers are determined by the inter-quartile range (Q3–Q1) for plotting. For each box and whisker plot, sample was drawn from the daily forecast values and stratified according to 4 quartile-based bins, namely the values within the ranges of minimum–Q1, Q1–Q2, Q2–Q3, and Q3–maximum, of aT2 or EHF for a selected period. The vertical box and whisker plots are for aT2 or EHF, and the horizontal box and whisker plots are for the corresponding circulation indices. (**a**,**b**) For 2-m temperature anomalies (aT2) and EHF, respectively, against the 4 circulation indices for SC-based-binning. (**c**,**d**) For WE-based-binning. Only the first and fourth quartile-based bins are shown in (**a**,**b**,**d**) for brevity, and denoted by blue and red colors, respectively. All 4 bins are shown in (**c**), with green and orange denote the second and third quartile-based bins. The solid and hollow boxes denote the forecast values for lead-week 1 and 4, respectively. The statistical analysis was conducted for the key period of 12 July–8 August, 2018.

The stratification of the forecasts based on positive and negative values was insufficient to reveal the magnitudes of the heatwave metrics and the circulation indices. We further stratified the forecasts by quartiles for each heatwave metric (aT2, T2-P90, or EHF) in either of the regions by using the following procedure. Based on the forecast values over each region, the MME forecasts were first grouped into four quartile bins according to the magnitudes of aT2, T2-P90, and EHF separately. Such data binning was based on the key period of 4 weeks: from 12 July to 8 August. We used four equal quartile bins, each of which contained 25% of the forecasts. For example, the first bin comprised the 25% of samples with the lowest aT2 in the MME forecasts. The circulation indices for the forecasts in each bin were then collected accordingly. We derived two sets of binned forecasts of various circulation indices (circulation indices stratified by quartiles for aT2, T2-P90, or EHF separately) based on the forecasted heatwave metrics of SC and WE, respectively. We term the two sets of binned forecasts as SC-based and WE-based data binning. The results of T2-P90 and EHF were similar; therefore, the results of aT2 and EHF are shown in Fig. 12. A further inspection of the data binning revealed that the upper quartiles of aT2 and EHF (bin containing the highest samples) are mostly forecasts from the second half of the key period, namely from 26 July to 8 August.

For the SC-based-binning, the binned aT2 and EHF both exhibited a linear relationship with the binned BL for lead weeks 1–4. Higher aT2 and EHF tended to occur with stronger BL, whereas lower aT2 and EHF occurred in the absence of blocking conditions (BL < 0). However, a linear relationship was not observed for the WE-based-binning, and no monotonic pattern was found relating the magnitudes of aT2 or EHF to blocking conditions. We further checked the corresponding binned forecasts and found that the upper quartiles of aT2 and EHF for the WE-based-binning were classified as SnWp (associated with positive AL-like pattern), whereas the medium quartiles were mostly classified as SpWp (associated with blocking situation and positive AL-like pattern). Strong positive AL phases contributed to the upper quartiles of aT2 and EHF of the SC-based-binning, this result reflected the findings from previous analyses of 500-hPa circulation patterns and height anomalies associated with SpWp. For weaker or negative AL, the relationships of AL with the magnitudes of aT2 and EHF in the SC-based-binning remain unclear. Apparent linear relationships were observed for the binned aT2 and EHF with binned AL from the WE-based-binning. Higher aT2 and EHF tended to occur with stronger positive AL, whereas lower aT2 and EHF occurred in the weaker or negative phase of AL. The result is consistent with the analyses of 500-hPa circulation patterns and height anomalies associated with SnWp. Accordingly, the MME forecasts accurately captured the influence of the magnitudes of positive phases of BL and AL on the warming amplitude for SC and WE, respectively. The magnitudes of the four bins for AZH exhibited apparent overlapping distributions (as comparing the boxes for lead weeks 1–2) from the SC-based-binning, indicating that this circulation index was not directly related to aT2 or EHF over SC. For the WE-based-binning, the binned AZH had a negative relationship with binned aT2 and EHF. Finally, binned NAO revealed no direct relationships with the binned aT2 or EHF for either SC or WE in the MME forecasts.

The interdependency between the BL, AL, and AZH was also captured in the MME forecasts for lead weeks 1–2. We recall the observed linear relationship: the positive correlations of AL and AZH with BL strongly affected the negative correlation between AL and AZH (Table 1). Because the data binning was conducted separately based on the forecasted heatwave metrics for SC and WE, the forecasts contained for each quartile bin were different between the two sets of binned forecasts. This resulted in different relationships between the binned forecasts according to the forecasted heatwave metrics of the two regions. The relationships of binned BL and AL with binned aT2 or EHF were positive for SC but not for WE. Because both BL and AL were positively correlated with the temperature variability of SC, the forecasted BL and AL exhibited a similar monotonic pattern of relationships when sorted by heatwave metrics for SC. This can be interpreted as the positive correlation of AL and BL. However, relationship between BL and AZH was less consistent from the SC-based-binning. The WE-based-binning did not clarify relationships of BL and AL because of the non-monotonic patterns between BL and the heatwave metrics of WE. This can be seen as a breakdown of the positive relationship between BL and AL, and thus a negative relationship between AL and AZH was emerged, in agreement with the observed linear relationship (Table 1).

## Summary

This study investigated the possible drivers and real-time extended-range prediction of the mid-July to early August 2018 heatwaves over northwestern Europe, with emphasis on the regional heatwave events over the SC and WE. The analysis was based on the ERA-Interim reanalysis data set and a multi-model ensemble of real-time forecasts from the S2S database. Three metrics were used to measure the persistent heatwave events namely warm spells, heatwave occurrence, and heatwave significance. The contributions of the following four circulation regimes to heatwave events over SC and WE were assessed: BL, AL, AZH, and NAO.

The persistent blocking regime was the most influential contributor to the 2018 heatwave over SC, and AL and NAO were secondary contributors to the heatwave with different effect direction. AL positively contributed to the heatwave, whereas NAO had the opposite effect. The major contributor to the heatwave over WE was AL. No linear relationships were found between the variability of AZH and the heatwaves for SC and WE. Causal relationships remained valid when the evolution of warm spells was considered.

The multi-model ensemble forecasts captured the evolution of warm spells over SC and WE for a lead time of up to 3 weeks. However, heatwave occurrence and significance were poorly predicted for the two regions. Prediction of heatwave occurrence with a lead time of 1–2 weeks was only possible when the heatwave event reached its mature stage. Variations in BL and AL were predicted 2 weeks in advance; these predictions contributed to successful prediction of warm spells for SC and WE, respectively, with lead time of 1–2. Although the variations of AZH and NAO were well captured in the forecasts, their contributions to the prediction of warm spells remain unclear.

The occurrence of prolonged heatwaves across northwestern Europe in the summer of 2018 have been attributed to the persistent and positive phase of NAO in the spring to summer in previous studies^5,9^. However, the variations in NAO had no positive effect on the heatwave occurrence on a daily or weekly timescale. Ferranti et al.^12^ investigated the role of model ability to capture transitions between flow patterns in predictions of high-impact weather at medium and extended ranges. They used the regime transitions of NAO and blocking associated with severe cold conditions over Europe as examples. During our analysis period, the variations of BL, AL, and AZH were interdependent. BL was positively correlated with AL and AZH, and these positive relationships strongly hindered the negative relationship between AL and AZH. This interdependence between the three circulation regimes was captured in the forecasts for the first two lead weeks. The prevalent positive phase of NAO was observed during the summer of 2018. The observation-based analyses revealed a positive relationship between monthly NAO and T2 anomalies over northwest Europe but a weak negative relationship between the daily NAO and T2 anomalies during this period. However, no linear relationship was found between NAO and T2 anomalies in the forecasts. Improvements in the model representation of short-term co-variations between AZH and NAO may increase the lead times for successful BL and AL prediction, leading to an increase in the lead times for the prediction of T2 anomalies.

## Data and methods

### Data

The Reanalysis of ERA-Interim (ERA-I) by the European Centre for Medium-Range Weather Forecasts (ECMWF)^35^ was used for analysis and forecast verification. The ERA-I daily 2-m temperature and 500-hPa geopotential height were interpolated onto the S2S grids. The temporal resolution was 6 h, from which we calculated the daily mean. The daily anomalies were calculated with respect to the period 1999–2010, same as the climate period used for the S2S models. Two teleconnection indices, namely NAO and SCA, were obtained from the NCEP/CPC (ftp://ftp.cpc.ncep.noaa.gov/wd52dg/data/indices/tele_index.nh). The two indices were defined based on the rotated EOF of geopotential height anomalies at 500 hPa^36^.

Forecasts from the subseasonal to seasonal (S2S) database^37^ were used. Individual models and their forecast ensembles are briefly described in Supplementary Table S1. This database contains 11 models, the ensemble sizes ranging from 51 members to four members. We used all 11 S2S models to form a set of multi-model ensembles (MMEs), which comprised 295 members. The used real‐time forecasts were initialized on Thursday in 10 of the 11 S2S models and on Wednesday in the Japan Meteorological Agency model. The daily forecast anomalies were first calculated for each model according to the model’s own climatology (July–August). The multi-model ensembles were then formed from all individual ensemble members. The periods of reforecast data differed between these models (Supplementary Table S1). The model climatologies were calculated as averages over the reforecasts for the period 1999–2010.

Our target period was early July to mid-August in 2018. We used all forecasts initialized from the nine initial dates available for the target period, namely 14 June, 21 June, 28 June, 5 July, 12 July, 19 July, 26 July, 2 August, and 9 August. The lead times for real-time forecast outputs were divided into four weeks, corresponding to days 1–7, 8–14, 15–21, and 22–28 from the initial dates. We term these four sets of lead times as lead weeks 1, 2, 3, and 4, respectively. The target period was divided into 6 weeks according to six of the nine initial dates.

## Methods

### Heatwave definition

Three metrics were used to characterizing the heatwave events in July 2018. By using the daily 2-m temperature (T2) field, we calculated the daily anomaly (aT2), the deviation from the 90th percentile value (T2-P90), and the excess heat factor (EHF)^38,39^. The aT2 value was calculated according to the monthly climatology, and T2-P90 was calculated according to the climatological 90th percentile threshold derived from the daily T2 of the respective month in a reference period. The reference period for the calculations of climatology, percentiles and EHF was 1999–2010. The daily EHF was calculated by combining the effects of heat severity and heat stress. The excess heat index of significance (${EHI}_{sig}$) measures the heat severity by combining a 3-day averaged temperature with a reference value; the excess heat index of acclimatization (${EHI}_{acc}$) measures the heat stress by combining a 3-day averaged temperature with the preceding 30-day mean temperature:

$${EHI}_{sig}=\frac{{T}_{i}+{T}_{i+1}+{T}_{i+2}}{3}-{T}_{90},$$

$${EHI}_{acc}=\frac{{T}_{i}+{T}_{i+1}+{T}_{i+2}}{3}-{T}_{mo},$$

$$EHF={EHI}_{sig}\mathrm{max}(1,{EHIT}_{acc}),$$

The temperature field can be a daily mean, a daily maximum, or a combination of maximum and minimum values. We used the daily mean T2 for the EHF calculation. The climatological 90th percentile threshold was used for the reference value. We used the monthly climatology for T2 instead of the preceding 30-day mean temperature value, and no difference was observed due to this replacement. In summary, aT2, T2-P90, and EHF were used to measure the duration of a warm spell, the occurrence of a heatwave event, and the severity of a heatwave, respectively.

### Circulation indices

#### Atmospheric blocking (BL) index

To identify a blocking situation, an objective blocking index^40,41^ was used. The meridional gradients of 500-hPa geopotential height, namely GHGS and GHGN, are computed for each longitude as follows:

$$GHGS = \frac{{\left[ {Z\left( {\phi _{0} } \right) - Z\left( {\phi _{S} } \right)} \right]}}{{\left[ {\phi _{0} - \phi _{S} } \right]}} ,$$

$$GHGN = \frac{{\left[ {Z\left( {\phi _{N} } \right) - Z\left( {\phi _{0} } \right)} \right]}}{{\left[ {\phi _{N} - \phi _{0} } \right]}} ,$$

where

$$\phi _{N} = 79.5^{ \circ }\, {\text{N}} \pm \Delta {\text{,}}$$

$${\phi}_{0}=60.0^{ \circ } \,\mathrm{N } \pm \Delta,$$

$${\phi}_{S}=40.0^{ \circ } \,\mathrm{N } \pm \Delta,$$

with $$\Delta = - 3.0^{ \circ } ,0.0^{ \circ } ,\;{\text{and}}\;3.0^{ \circ }$$.

The values of the aforementioned latitudes (${\phi}_{X}$) and increments ($\Delta$) differ slightly from those of D’ Andrea et al.^40^ and Tibaldi and Molteni^41^ because of the S2S grids. A specific longitude is defined as being locally blocked if $GHGS>0\text{ and }GHGN<-5\mathrm{ m}/^{ \circ }\mathrm{N}$ for at least one value of $\Delta$. We defined the longitudinal limits to be 0°–40°E for the blocking-prone European sector. The sector was defined as blocked if three or more adjacent longitudes within its limits were blocked according to the local blocked index. GHGS strength was sufficient for representing the blocking activities in the summer of 2018. Therefore, we used GHGS strength alone to assess model behavior; we hereafter refer to GHGS as BL for brevity.

#### Atlantic Low (AL) index

We adopted a center of action approach to construct the time series for AL based on the spatial pattern provided by Cassou et al.^25^ (Fig. 1 b in their paper). We calculated the daily difference in the area average of normalized geopotential height anomalies at 500 hPa between two areas: 3°–9°E and 18°–24°W for the latitudinal zone of 48°–54°N.

#### Azores High (AZH) index

The southern node of the NAO dipole almost overlaps with the activity region of the North Atlantic subtropical high, also known as the Azores High. To measure the variability of the Azores High, we calculated the regional mean of the normalized geopotential height anomalies at 500 hPa over the area 24°–54°W and 30°–45°N. Substantial variability in the central of the Azores High during summer was observed through a comparison of different study periods^42,43^. We adapted the area for the variations in the center of the summertime Azores High determined by Falarz^42^. Linear regressions of 2-m temperature anomalies and 500-hPa geopotential height anomalies in July onto the monthly AZH index revealed similar spatial patterns to those of CPC-obtained monthly SCA index (Supplementary Fig. S2).

#### North Atlantic Oscillation (NAO) index

To define the NAO index, we adapted the meridional gradient method^44,45^ to calculates the zonal mean difference of normalized 500-hPa geopotential height over the longitudinal sector 70°W–10°W. The normalized geopotential height anomalies were first calculated over the latitudinal ranges of 36°–45°N and 66°–75°N. Other definitions of NAO include the station-based method, in which the difference in normalized sea-level pressure anomalies is calculated between a southern station and a northern station^46,47^, and the PC-based method, in which principle component analysis is applied to gridded climate datasets^29,36^. We adopted the meridional gradient of the zonal averaged geopotential height anomalies for the following reasons: (1) the station-based method results in considerable noise from transient and localized signals, and (2) the use of gridded data in the PC-based method results in a nonstationary loading pattern. These two shortcomings compromise the accuracy of comparisons between the S2S model outputs and the reanalysis. The linear regressions of 2-m temperature anomalies and 500 hPa geopotential height anomalies in July onto our monthly NAO index revealed similar spatial patterns to those of the monthly NAO index obtained from the CPC (Supplementary Fig. S2).

### Statistical approaches

For the reanalysis data, the relationships of the 2-m temperatures and 500 hPa geopotential heights with circulation indices during heatwaves were examined using bivariate correlation, partial correlation, and multiple linear regression. All these analyses were conducted for the period from July 1 to August 31, 2018. Statistical significance was determined using a two-tailed Student’s *t* test. For the S2S forecasts, we analyzed the distributions of all the ensemble members for the MME. The majority of the results were obtained from quartile statistics.

## Supplementary information

Supplementary Information.
