# Supplementary material for: The 2018 summer heatwaves over northwestern Europe and its extended-range prediction
Source: Sci Rep. 2020 Nov 6;10:19283. doi: 10.1038/s41598-020-76181-4 (PMC7648626; doi:10.1038/s41598-020-76181-4)
Supplement: Supplementary file 1 — Supplementary Information. [file 41598_2020_76181_MOESM1_ESM.pdf]

Supplements for:

The 2018 summer heatwaves over northwestern Europe and its extended-range prediction

Mien-Tze Kueh[1], **and Chuan-Yao Lin**[1,*]

[1]Research Center for Environmental Changes, Academia Sinica, Taipei, Taiwan
*Chuan-Yao Lin (yao435@rcec.sinica.edu.tw)

This file contains supplementary figures and table:

Figure S1
Figure S2
Figure S3
Figure S4
Figure S5

Table S1

Supplementary Figure S1

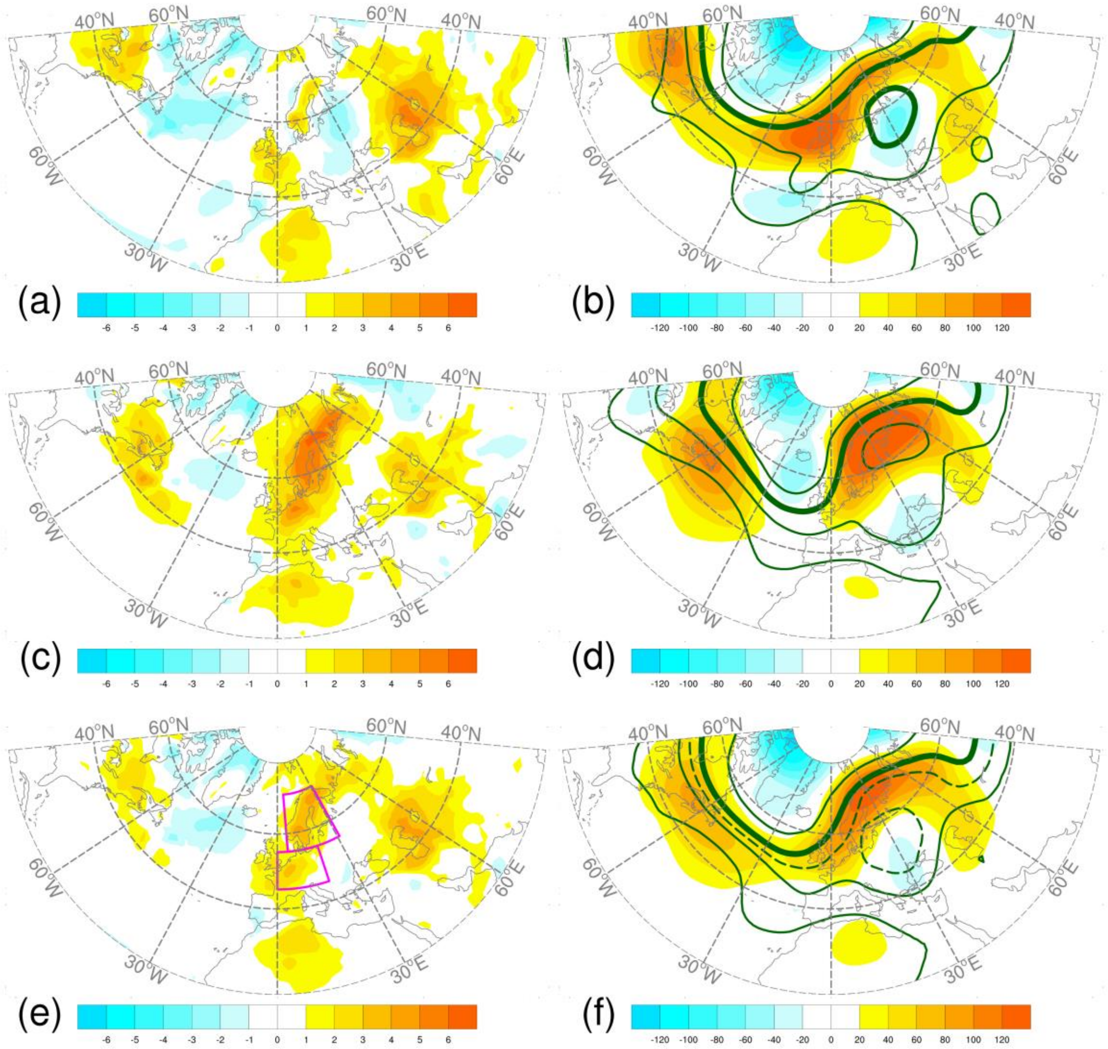

Figure S1. 2018 (Left panels) 2-m temperature anomalies (shading, °C) and (Right panels) 500-hPa geopotential height anomalies (shading, m) and absolute 500-hPa geopotential height (contour, m). The solid contours are 5600, 5700, and 5800 m, and dashed contour is 5750 m. Anomalies are relative to the period of 1999—2010. (a) and (b) for the mean of the first half (1-15) of July, (c) and (d) for the mean of the second half (16-31) of July. (e) and (f) for the mean of July, and are the same as Figure 1 a and b. Boxes show the area used to define Scandinavia (SC) and Western Europe (WE). The maps were generated using software NCAR Command Language (https://www.ncl.ucar.edu/)[32] with the built-in Ncarg4_0 database.

Supplementary Figure S2

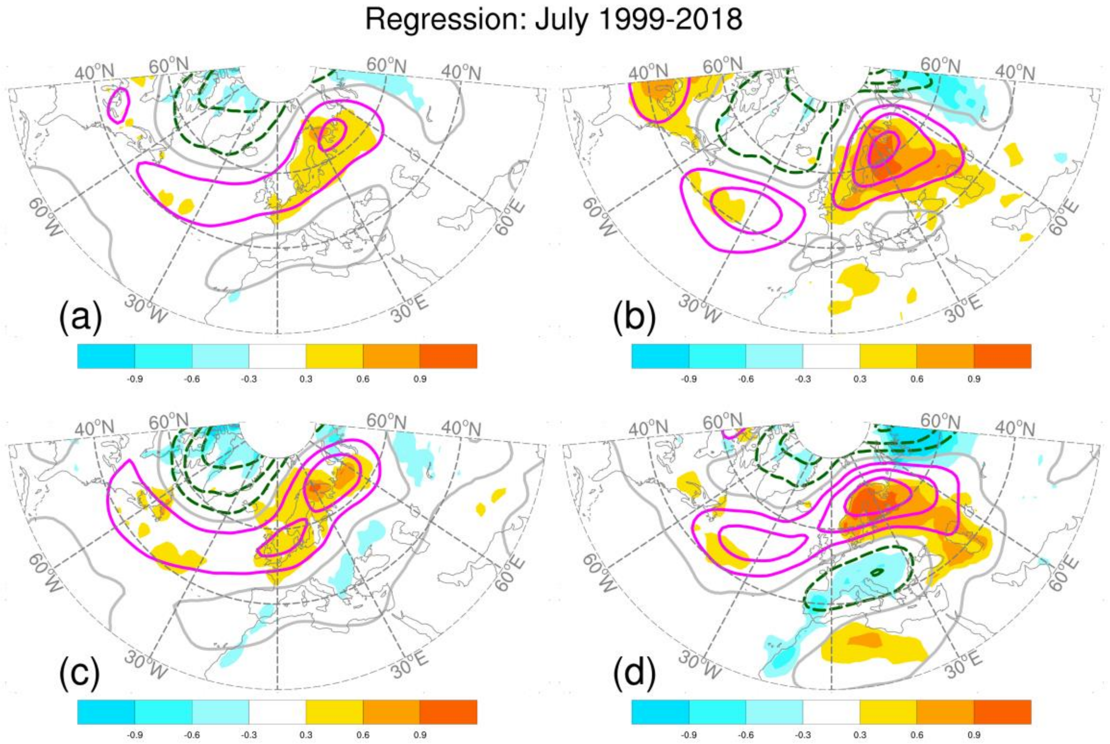

Figure S2. Regressions of July 2-m temperature anomalies (shading, °C) and 500-hPa geopotential height anomalies (shading, m) onto the monthly circulation indices (a) NAO calculated in this study, (b) AZH, (c) NAO from CPC and (d) SCA from CPC. Anomalies are relative to the period of 1999—2010. NAO=North Atlantic Oscillation, AZH=Azores High, SCA=Scandinavia Pattern. Contour interval is 2; solid and dashed contours for positive and negative correlation coefficients, respectively, and gray color for zero contours. The linear regression analyses were conducted over the period of July 1999—2018. The temporal correlation coefficient between the NAO from and that calculated in this study is 0.88 and the correlation is statistically significant at the 0.001 level with a two-tailed Student *t*-test. The maps were generated using software NCAR Command Language (https://www.ncl.ucar.edu/)[32] with the built-in Ncarg4_0 database.

Supplementary Figure S3

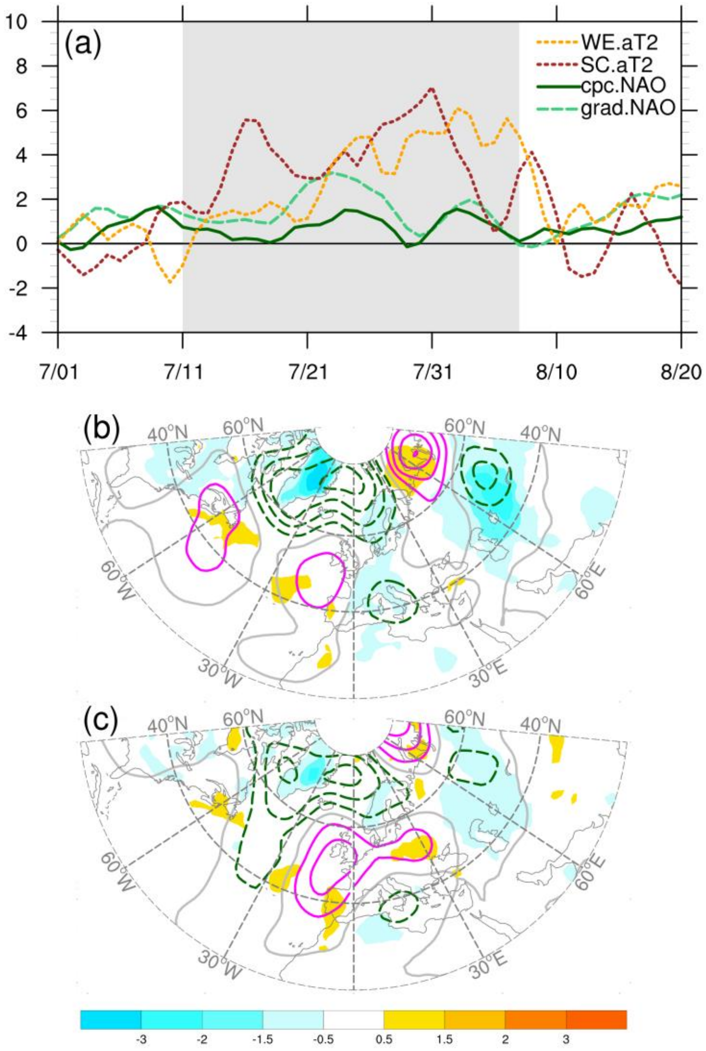

Figure S3. Top panel: (a) Time series of aT2 over SC and WE, and daily NAO derived in this study and obtain from CPC during period of 1 July to 20 August, 2018. Gray shading region covers the period of 12 July to 8 August, 2018. Central and bottom panels: Linear regression of daily anomalies of 2-m temperature (shading, °C) and 500-hPa geopotential height (contour, m) onto the standardized circulation indices (b) NAO from this study, and (c) NAO from CPC. Solid and dashed contours for positive and negative regression coefficients, respectively, and gray contours for zero. Contour interval is 150 m per standard deviation change in index for 500-hPa geopotential height anomalies. Contour interval for 2-m temperature anomalies is denoted by label bars, the units is °C per standard deviation change in index. The linear regression analyses were conducted for period of 1 July to 31 August, 2018. The temporal correlation coefficient between the NAO from CPC and that calculated in this study is 0.60 and is statistically significant at the 0.01 level with a two-tailed Student *t*-test. The maps were generated using software NCAR Command Language (https://www.ncl.ucar.edu/)[32] with the built-in Ncarg4_0 database.

Supplementary Figure S4

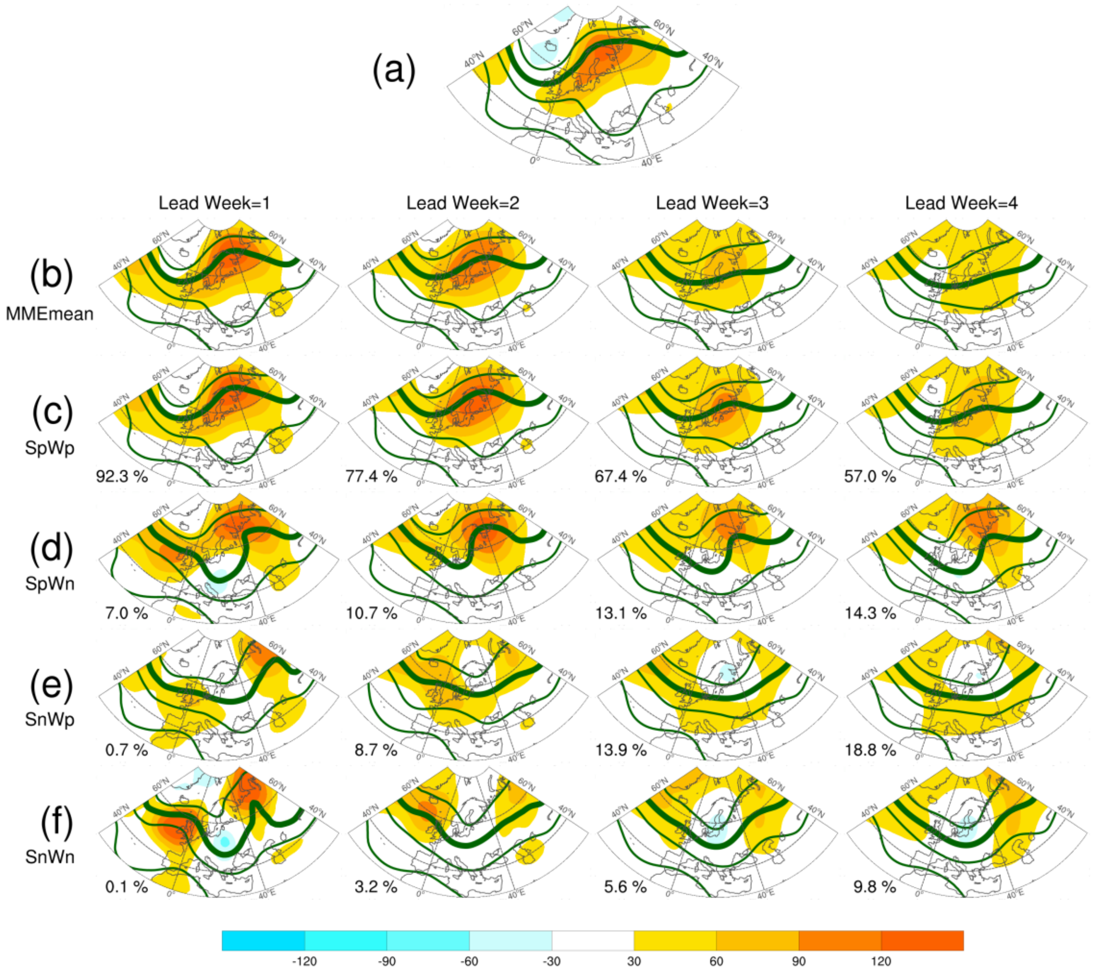

Figure S4. The 500-hPa geopotential height anomalies (shading, m) and absolute 500-hPa geopotential height (contour, m) for the key period from 12 July to 8 August, 2018. (a) is for the ERA-I, (b)-(f) are ensemble mean calculated according to the four groups of probabilities based on the forecasts of aT2. Numeral on each panel indicates the proportions of each possibility for each lead week, also shown in Figure 10. The solid contours are 5600, 5700, 5800, and 5900 m. Anomalies are relative to the period of 1999—2010. The maps were generated using software NCAR Command Language (https://www.ncl.ucar.edu/)[32] with the built-in Ncarg4_0 database.

Supplementary Figure S5

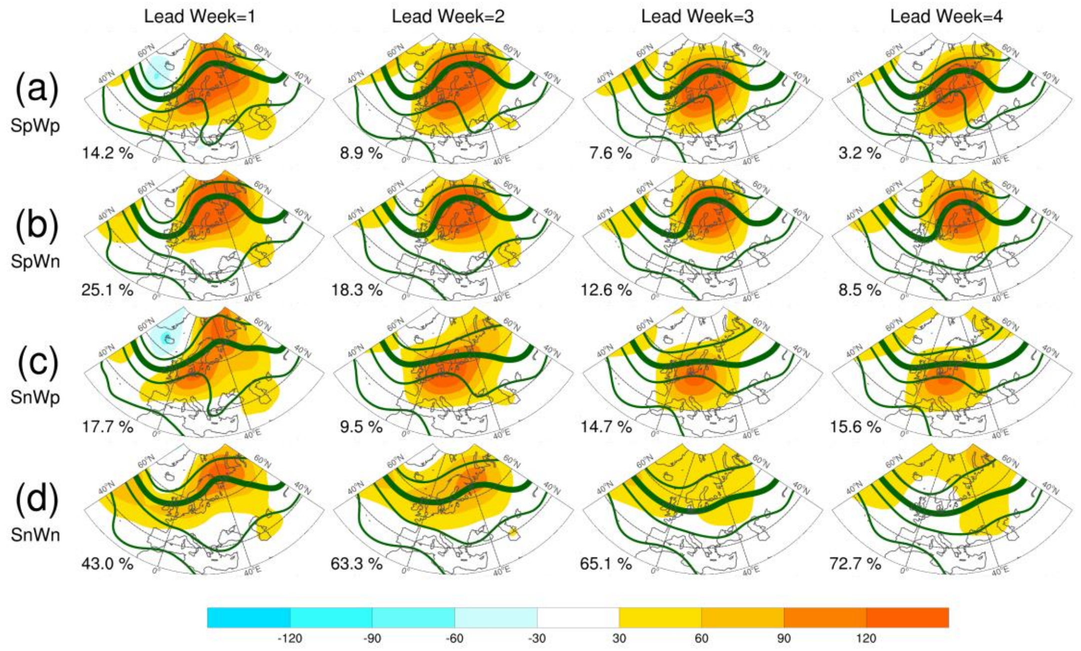

Figure S5. Same as in Figure S4 c-f, but for the four groups of probabilities based on the forecasts of EHF.

Supplementary Table S1

Table S1. Brief description of S2S models used for the compilation of the multi-model ensemble in this study. In Figure 6, the box and whisker plots for the 11 models within each 7-day stage, from left to right, are arranged as the descending order in this table. Data grid: BoM= T46 (144x75), other models= 1.5x1.5.

Model	Institute	Ensemble members*	Forecast length (days)	Realtime forecast frequency	Reforecast frequency	Reforecast period
BoM	*Bureau of Meteorology*	33 [33]	62	Sun Thu	Six per month	1981-2013
CMA	*China Meteorological Administration*	4 [4]	60	Daily	Daily	1994-2014
ECCC	*Environmental and Climate Change Canada*	21 [4]	32	Thu	Thu	1995-2014
ECMF	*European Centre for Medium-Range Weather Forecasts*	51 [11]	46	Mon Thu	Mon Thu	Past 20 years
HMCR	*Hydrometeorological Center of Russia*	20 [10]	61	Thu	Thu	1985-2010
ISAC	*Institute of Atmospheric Sciences and Climate*	41 [5]	31	Thu	Every 5 days	1981-2010
JMA	*Japan Meteorological Agency*	50 [5]	32.5	Wed	Three per month	1981-2012
KMA	*Korea Meteorological Administration*	4 [3]	60	Daily	Four per month	1991-2010
MFr	*Météo-France*	51 [15]	32	Thu	Twice per month	1993-2014
NCEP	*National Centers for Environment Prediction*	16 [4]	44	Daily	Daily	1999-2010
UKMO	*UK Met-Office*	4 [7]	60	Daily	Four per month	1993-2015

- Ensemble members: Real-time [Reforecast]
